# Immunogenic Cell Death of Breast Cancer Stem Cells Induced by an Endoplasmic Reticulum‐Targeting Copper(II) Complex

**DOI:** 10.1002/cbic.202000553

**Published:** 2020-09-22

**Authors:** Pooja Kaur, Alice Johnson, Joshua Northcote‐Smith, Chunxin Lu, Kogularamanan Suntharalingam

**Affiliations:** ^1^ Department of Immunology and Inflammation Imperial College London The Hammersmith Hospital Du Cane Road London W12 0NN UK; ^2^ School of Chemistry University of Leicester University Road Leicester LE1 7RH UK; ^3^ College of Biological, Chemical Sciences and Engineering Jiaxing University 56 South Yuexiu Road Jiaxing 314001 Zhejiang P. R. China

**Keywords:** bioinorganic chemistry, cancer stem cells, copper, endoplasmic reticulum, immunogenic cell death

## Abstract

Immunogenic cell death (ICD) offers a method of stimulating the immune system to attack and remove cancer cells. We report a copper(II) complex containing a Schiff base ligand and a polypyridyl ligand, **4**, capable of inducing ICD in breast cancer stem cells (CSCs). Complex **4** kills both bulk breast cancer cells and breast CSCs at sub‐micromolar concentrations. Notably, **4** exhibits greater potency (one order of magnitude) towards breast CSCs than salinomycin (an established breast CSC‐potent agent) and cisplatin (a clinically approved anticancer drug). Epithelial spheroid studies show that **4** is able to selectively inhibit breast CSC‐enriched HMLER‐shEcad spheroid formation and viability over non‐tumorigenic breast MCF10 A spheroids. Mechanistic studies show that **4** operates as a Type II ICD inducer. Specifically, **4** readily enters the endoplasmic reticulum (ER) of breast CSCs, elevates intracellular reactive oxygen species (ROS) levels, induces ER stress, evokes damage‐associated molecular patterns (DAMPs), and promotes breast CSC phagocytosis by macrophages. As far as we are aware, **4** is the first metal complex to induce ICD in breast CSCs and promote their engulfment by immune cells.

## Graphical abstract

**Find me, eat me**: Cancer stem cells (CSCs) are heavily linked to patient relapse, so targeting them could improve treatment. One method of inducing a tumour‐targeting immune response is immunogenic cell death (ICD), in which dying cancer cells stimulate tumour‐associated immune cells to seek and remove them. Here we report the first metal complex to induce ICD in CSCs.

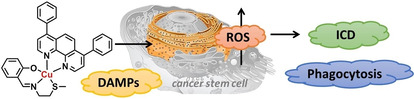

## 1 Introduction

Cancer relapse and metastasis, the leading cause of cancer associated deaths, is strongly linked to the existence of cancer stem cells (CSCs), a small subpopulation of cancer cells defined by their ability to self‐renew, differentiate, and form secondary tumours.^[1]^ CSCs evade conventional chemotherapy and radiotherapy as these treatments tend to specifically target fast growing cancer cells, and CSCs, due to their stem cell‐like quiescent nature, divide more slowly.^[2]^ After surviving treatment, CSCs are able to regenerate the original tumour and/or produce invasive cancer cells that can colonise distant organs.^[3]^ The clinical implication of CSCs means that cancer treatments must have the ability to remove heterogeneous tumour populations in their entirety, including CSCs, otherwise CSC‐mediated relapse could occur. Potential CSC therapeutic targets such as cell surface markers, deregulated signalling pathways, and components within the microenvironments in which they reside have been identified but there is still no clinically approved drug that can fully remove CSCs.^[4]^ Immunotherapy, where the immune system is stimulated to destroy tumours, has recently emerged as a viable alternative to conventional therapies, and could provide long‐term therapeutic outcomes.^[5]^ Topical research suggests immunotherapeutic strategies that target CSCs may improve the efficacy of cancer treatment when used in combination with traditional cytotoxic therapies.^[6]^ Therefore, the development of new immuno‐chemotherapeutic agents (such as metal complexes) capable of reducing tumour mass by cytotoxic mechanisms and removing residual CSCs by immunological activation could revolutionise oncology.

One of the methods by which existing chemotherapeutics induce a tumour‐targeting immune response is through immunogenic cell death (ICD), where the dying cancer cells stimulate tumour‐associated (TA) immune cells to actively seek and destroy them by exposing “find me” and “eat me” protein signals.^[7]^ CSCs that have undergone ICD can potentially act as “vaccines” and initiate a robust immune response. The induction of ICD in CSCs is a surprisingly underexplored arm of immunotherapy. The ability of small molecules (classified as type II ICD inducers) to induce ICD of bulk cancer cells is allied to their ability to evoke focused reactive oxygen species (ROS)‐mediated endoplasmic reticulum (ER) stress.^[8]^ Compounds that induce collateral ER stress (type I ICD inducers) are less effective immunogenic agents. To date, only a handful of type II ICD inducers have been identified and only a few examples contain a metal.^[9]^ Of the clinically approved anticancer platinum(II) agents (cisplatin, carboplatin, and oxaliplatin), only oxaliplatin has been reported to induce ICD (in bulk colon cancer cells).^[10]^ Cisplatin was reported to induce ICD in bulk osteosarcoma cells only when administered with thapsigargin, a sarco/endoplasmic reticulum calcium ATPase (SERCA) inhibitor.^[11]^ ER‐targeting platinum(II)‐N‐heterocyclic carbene and platinum(II)‐aminophosphonate complexes were identified to induce all the hallmarks of ICD in bulk colon and urinary bladder cancer cells, respectively.^[12]^ Platinum(IV) complexes comprising of oxaliplatin and *rac*‐2‐(2‐propynyl)octanoato (a histone deacetylase inhibitor) or SZU101 (a toll‐like receptor 7 agonist) induced ICD in mice bearing highly aggressive CT26 colon or 4T1 breast carcinoma, respectively.^[13]^ In both instances, ICD was demonstrated by the detection of activated cytotoxic CD8+ T lymphocytes within the tumour mass.^[13]^ Sodium *trans*‐[tetrachloridobis(1H‐indazole)‐ruthenate(III)] (also known as KP1339/IT‐139), a clinically investigated drug, was shown to induce ICD in bulk colon cancer spheroid models.^[14]^ All of the studies on ICD‐inducing metal complexes reported thus far have focused on bulk cancer cells, therefore the impact of ICD‐inducing metal complexes on CSCs is completely unexplored.

Copper, an endogenous metal (of which humans have 1.4‐2.1 mg/kg of body weight), has efficient redox‐cycling properties under physiological conditions when coordinated to the appropriate ligands.^[15]^ Thus copper complexes can be employed to efficiently elevate ROS levels inside cells.^[15]^ We have previously shown that breast CSCs are more susceptible to ROS elevation by copper(II) complexes than other cell types (including bulk breast cancer cells and normal cells).^[16]^ Here, we present a series of copper(II) complexes, **1**–**4** and their potency towards breast CSCs, and moreover, show that the 4,7‐diphenyl‐1,10‐phenanthroline‐bearing complex, **4** is able to induce oxidative ER stress and ICD in breast CSCs *in vitro*. To the best of our knowledge, **4** is the first metal complex to evoke ICD hallmarks in CSCs.

## 2 Results and Discussion

**Figure 1:**
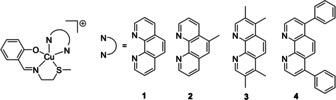
Chemical structures of the copper(II) complexes, **1**–**4** investigated in this study. The charged copper(II) complexes were all isolated as hexafluorophosphate salts.

To evoke ICD in CSCs, compounds are expected to localise in the ER and generate ROS. With this in mind, we developed a series of copper(II) complexes with a Schiff base ligand, **L^1^** (a proven ROS mediator once coordinated to copper, see Figure S1 in the Supporting Information for chemical structure)[^15^, ^16c^, ^17^] and various lipophilic polypyridyl ligands (known to facilitate localisation in the lipid dense ER).^[18]^ The copper(II) complexes, **1**–**4** used in this study are shown in Figure 1. The complexes, **1**–**4** were prepared by reacting equimolar amounts of the appropriate polypyridyl ligand with copper(II) nitrate hydrate in methanol, followed by the addition of the Schiff base ligand, **L^1^** (synthesised according to reported protocols)[^16c^, ^19^] and excess sodium hexafluorophosphate. The complexes were isolated in low to reasonable yields (12–68 %) as green‐blue solids and characterised by high‐resolution ESI mass spectrometry, IR spectroscopy, and elemental analysis (see the Supporting Information). Distinctive molecular ion peaks corresponding to **1**–**4** with the appropriate isotopic pattern were observed in the HRMS (ESI) (*m*/*z* 468.0421 [**1**‐PF_6_‐H+CH_3_OH]^+^; 451.0773 [**2**‐PF_6_]^+^; 493.1253 [**3**‐PF_6_]^+^; 589.1256 [**4**‐PF_6_]^+^, Figures S2–S5). The IR spectra for **1**–**4** displayed C=N_imine_ bands between 1604–1619 cm^−1^ indicating the presence of the imine functionality associated to **L^1^** (the C=N_imine_ band for **L^1^** appears at 1628 cm^−1^; Figure S6). Furthermore, the IR spectra for **1**–**4** did not display a broad O−H stretch (Figure S6), thus supporting the complexation of **L^1^** to the copper centre (as depicted in Figure 1). The purity of **1**–**4** was established by elemental analysis. [Cu(**L^1^**)Cl] (see Figure S1 for chemical structure) was also prepared to serve as a control compound–a copper(II) complex without an ER‐targeting polypyridyl ligand. The synthetic protocol and full characterisation of [Cu(**L^1^**)Cl] is reported in the Supporting Information.

**Figure 2:**
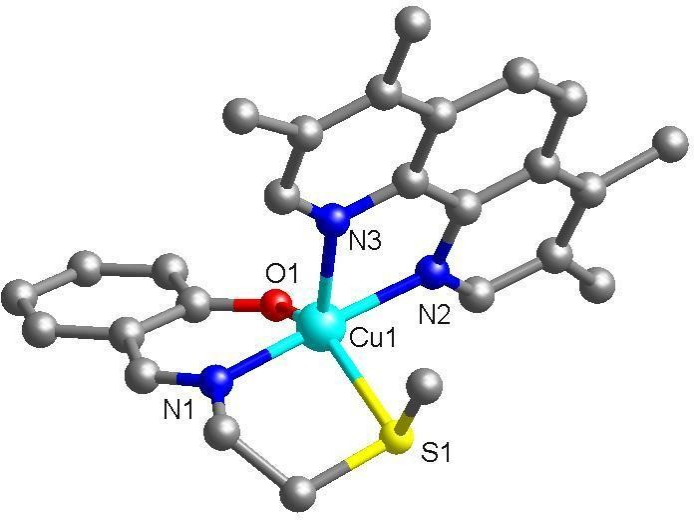
X‐ray structure of the copper(II) complex, **3** comprising **L^1^** and 3,4,7,8‐tetramethyl‐1,10‐phenanthroline. Ball and stick representation, C in grey. H atoms and the hexafluorophosphate counter anion have been omitted for clarity.

Single crystals (blue blocks) of **3** suitable for X‐ray diffraction studies were obtained by slow evaporation of a methanol solution of **3** (CCDC 1943504, Figure 2, Table S1). Selected bond distances and angles data are presented in Table S2. The complex exhibits a distorted square‐based pyramidal geometry with the copper centre coordinated to **L^1^** in a tridentate manner and 3,4,7,8‐tetramethyl‐1,10‐phenanthroline in a bidentate manner. The Cu−N_imine_ (1.9408(18) Å), Cu−N_polypyridyl_ (2.0017(17) and 2.1797(18) Å), Cu−S (2.4589(7) Å), and Cu−O (1.9122(16) Å) bond lengths are consistent with bond parameters observed for related five‐coordinate copper(II) complexes.[^19^, ^20^] Within the CuN_2_OS basal plane, the N(1)−Cu−N(2) angle is 177.29(8)° and the O(1)−Cu−S(1) angle is 141.93(6)°; this is consistent with a distorted square‐based pyramidal geometry.

The lipophilicities of **1**–**4** were determined by measuring the extent to which it partitioned between octanol and water, *P*. The experimentally determined log *P* values varied from 0.73 to 2.01 (Table S3). The hydrophobic nature of **1**–**4** suggests that the copper(II) complexes should be readily taken up by cells. UV/Vis spectroscopy studies were carried out to assess the stability of the copper(II) complex, **1** taken as a representative member of the copper(II) complexes, in biologically relevant solutions. The UV/Vis absorption bands of the copper(II) complex, **1** (25 μM) in PBS/DMSO (200 : 1) and mammary epithelial cell growth medium (MEGM):DMSO (200 : 1) remained largely unaltered over the course of 24 h at 37 °C suggestive of stability (Figures S7 and S8).

The antiproliferative properties of the copper(II) complexes **1**–**4** against breast CSC‐depleted (HMLER) and breast CSC‐enriched (HMLER‐shEcad) cells was determined and compared to salinomycin (an established breast CSC‐potent agent) and cisplatin (a clinically approved platinum(II)‐based anticancer drug). The IC_50_ values were determined from dose–response curves (Figures S9–S12) and are summarised in Table 1. The complexes, **1**–**4** displayed sub‐micro‐ or micromolar potency towards both HMLER and HMLER‐shEcad cells. There was a clear correlation between cytotoxicity and the of the polypyridyl ligand present, with bulkier ligands endowing higher lipophilicity (see log *P* values in Table S3), and HMLER and HMLER‐shEcad cell potency. The most potent complex within the series, **4** displayed 13‐fold and 18‐fold greater potency (*p* <0.05, n=18) for CSC‐enriched HMLER‐shEcad cells than salinomycin and cisplatin, respectively (Table 1).[^16a^, ^21^] Control studies showed that [Cu(**L^1^**)Cl] was nontoxic towards HMLER and HMLER‐shEcad cells (IC_50_>100 μM), indicating that the cytotoxicity of **1**–**4** towards bulk breast cancer cells and breast CSCs is likely to result from the intact copper(II) complexes, containing both the Schiff base, **L^1^** and the corresponding polypyridyl ligand (Figure S13, Table 1).

**Table 1:** IC_50_ values of the copper(II) complexes, **1**–**4** and [Cu(**L^1^**)Cl], cisplatin, and salinomycin against HMLER cells, HMLER‐shEcad cells, and HMLER‐shEcad spheroids.

Compound	IC_50_ [μM]		
	HMLER^[a]^	HMLER‐shEcad^[a]^	Spheroid^[b]^
**1**	1.14±0.02	1.52±0.22	9.57±0.06
**2**	0.78±0.05	0.98±0.10	9.64±0.03
**3**	0.75±0.02	0.81±0.02	2.37±0.01
**4**	0.21±0.01	0.32±0.02	0.54±0.01
[Cu(**L^1^**)Cl]	>100	>100	85.78±0.17
cisplatin ^[c]^	2.57±0.02	5.65±0.30	13.50±2.34
salinomycin ^[c]^	11.43±0.42	4.23±0.35	18.50±1.50

[a] Determined after 72 h of incubation (mean of three independent experiments±SD). [b] Determined after 5 days′ incubation (mean of three independent experiments±SD). [c] Reported in refs. [16a], [17] and [21].

**Figure 3:**
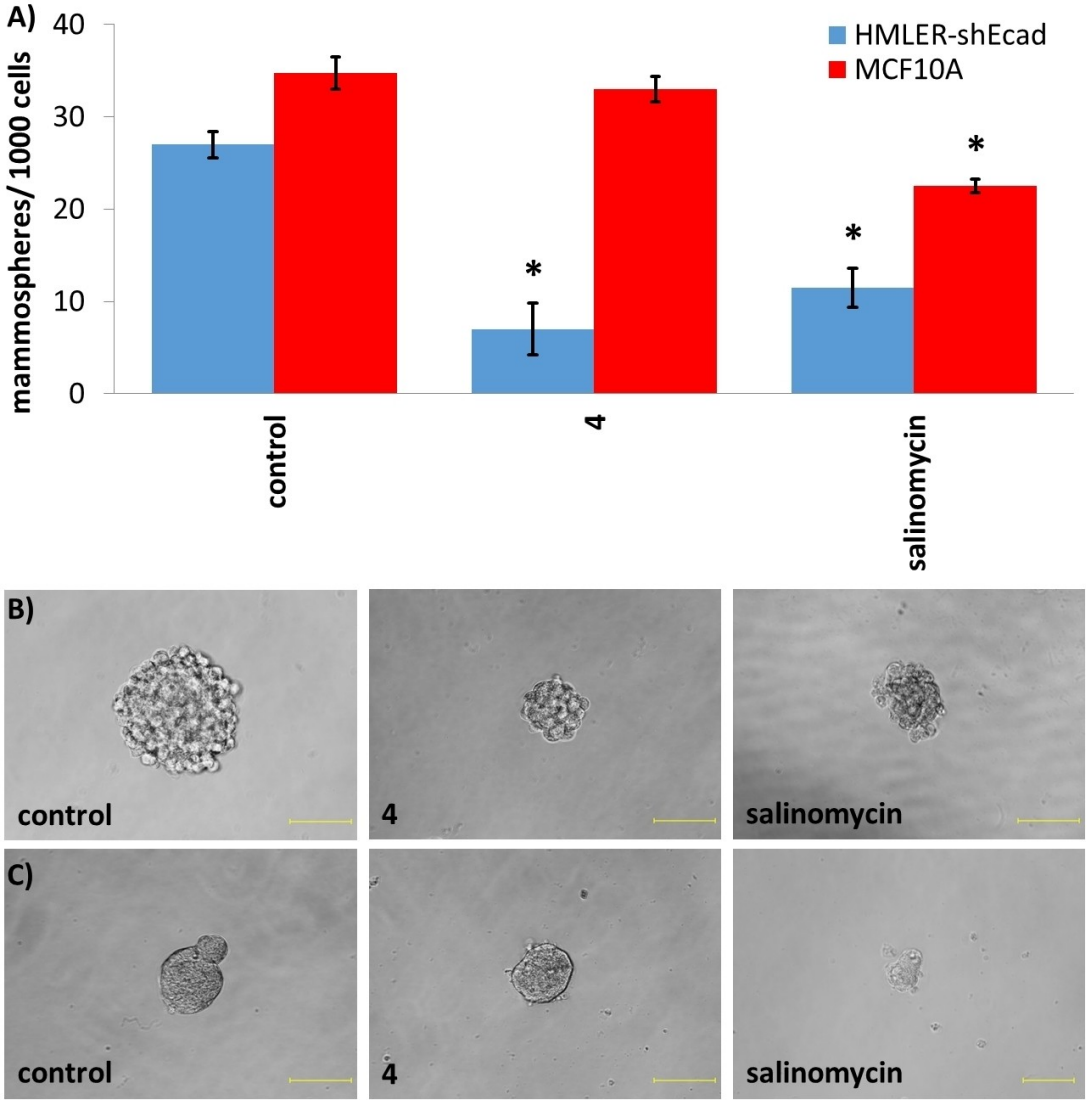
A) Quantification of spheroid formation with HMLER‐shEcad and MCF10 A cells untreated and treated with **4** or salinomycin at their respective IC_20_ values for 5 days. Error bars=SD and Student t‐test, * *p* <0.05. Representative bright‐field images (×20) of B) HMLER‐shEcad and C) MCF10 A spheroids in the absence and presence of **4** or salinomycin at their respective IC_20_ values for 5 days. Scale bars: 100 μm.

Epithelial breast cells (cancer and nontumorigenic), when grown in serum‐free media under low‐attachment conditions, are capable of forming three‐dimensional spheroids.^[22]^ The ability of a given compound to inhibit spheroid formation from single cell suspensions (with respect to number, size, and viability) is often used as a marker for *in vivo* potency, given that three‐dimensional systems are more representative of organs and tumours than monolayer cell cultures. The ability of **1**–**4** to inhibit breast CSC‐enriched HMLER‐shEcad spheroid formation (at a nonlethal dose) was assessed using an inverted microscope. The addition of **1**–**4** (IC_20_ value for 5 days) and salinomycin (IC_20_ value for 5 days, positive control) to single cell suspensions of HMLER‐shEcad cells significantly (*p* <0.05) decreased the number and size of HMLER‐shEcad spheroids formed (Figures 3A and B, S14 and S15). [Cu(**L^1^**)Cl] had little effect on the number of HMLER‐shEcad spheroids formed, however, it substantially decreased the size of HMLER‐shEcad spheroids formed (Figures S14 and S16). Notably, the HMLER‐shEcad spheroid inhibitory effect of **4** (74 % decrease in number of HMLER‐shEcad spheroids formed compared to the untreated control) was greater than that observed for salinomycin under identical conditions (57 % decrease in number of HMLER‐shEcad spheroids formed compared to the untreated control, Figure S14). To gauge the ability of **1**–**4** to decrease HMLER‐shEcad spheroid viability, the colorimetric resazurin‐based reagent, TOX8 was employed. The copper(II) complexes, **1**–**4** exhibited sub‐micro‐ or micromolar potency towards HMLER‐shEcad spheroids (Figure S17, Table 1). Strikingly, the IC_50_ value for **4** (0.54±0.01 μM) was 34 and 25 times lower than that reported for salinomycin and cisplatin, respectively, under identical conditions.[^17^, ^21^] [Cu(**L^1^**)Cl] displayed significantly (*p*<0.05) lower potency for HMLER‐shEcad spheroids than **1**–**4**, (Figure S17, Table 1) indicating that the intact copper(II) complexes containing both the Schiff base, **L^1^** and the corresponding polypyridyl ligand are responsible for the observed activities with HMLER‐shEcad spheroids.

The addition of the most potent copper(II) complex, **4** (IC_20_ value for 5 days) to single cell suspensions of nontumorigenic breast epithelial MCF10 A cells did not significantly (*p*=0.20) change the number or size of MCF10 A spheroids formed (Figure 3A and C). In contrast, treatment with salinomycin under the same conditions resulted in a significant (*p*<0.05) decrease in the number (35 % decrease) and size of MCF10 A spheroids formed (Figure 3A and C). This is similar to the result obtained with HMLER‐shEcad spheroids (Figures S14 and S15). Spheroid viability studies showed that **4** killed nontumorigenic MCF10 A spheroids (IC_50_=1.89±0.03 μM) with 3.5‐fold lower potency than CSC‐enriched HMLER‐shEcad spheroids (Figure S18). Salinomycin on the other hand, killed MCF10 A spheroids (IC_50_=14.90±0.50 μM) similarly to HMLER‐shEcad spheroids under identical conditions (Figure S19).^[22b]^ Overall the epithelial spheroid studies show that **4** is able to selectively inhibit breast CSC‐enriched HMLER‐shEcad spheroid formation and viability over nontumorigenic breast epithelial MCF10 A spheroids.

As complex **4** exhibited the highest activity against breast CSCs in monolayer and three‐dimensional cell culture systems, and contained 4,7‐diphenyl‐1,10‐phenanthroline (a moiety that is present in many ER stress‐inducing metal complexes),^[23]^ we investigated its ability to function as a type II ICD inducer in breast CSCs. Type II ICD inducers are expected to generate ROS in the ER, leading to ER stress. Therefore, we determined the ability of **4** to enter breast CSCs and localise in their ER using inductively coupled plasma mass spectrometry (ICP‐MS). HMLER‐shEcad cells treated with **4** (5 μM for 24 h at 37 °C) displayed a relatively large amount of copper (569.3±17.6 ppb of Cu/million cells), indicative of effective cell uptake. A substantial amount of internalised **4** was also detected in the ER (103.0±2.2 ppb of Cu/million cells; Figure S20). This suggests that **4** can enter the ER in breast CSCs and possibly cause ER stress. Control studies with HMLER‐shEcad cells treated with [Cu(**L^1^**)Cl] (5 μM for 24 h at 37 °C) showed that although [Cu(**L^1^**)Cl] enters HMLER‐shEcad cells at appreciable levels (101.7±3.4 ppb of Cu/million cells), the amount reaching the ER was nine times lower (12.0±0.3 ppb of Cu/million cells) than **4** (Figure S20). This shows that the 4,7‐diphenyl‐1,10‐phenanthroline ligand in **4** plays an important role in enhancing whole cell uptake and ER localisation in breast CSCs.

The ability of **4** to elevate intracellular ROS levels was probed using 6‐carboxy‐2’,7’‐dichlorodihydrofluorescein diacetate (DCFDA), a well‐established ROS fluorescent indicator. HMLER‐shEcad cells treated with **4** (IC_50_ value) displayed a time‐dependent increase in ROS levels up to 16 h exposure (20‐43 % increase in detectable ROS levels compared to untreated cells, *p*<0.05; Figure S21). Prolonged exposure (24 and 48 h) did not significantly increase intracellular ROS levels (Figure S21). Similar time‐dependent ROS production has been reported for other metal complexes that induce cell death by ROS‐dependent mechanisms.[^16c^, ^24^] Cytotoxicity studies in the presence of *N*‐acetylcysteine (NAC), a ROS scavenger (2.5 mM, 72 h) showed that the potency of **4** towards HMLER‐shEcad cells decreased (IC_50_ value increased from 0.32±0.02 μM to 0.49±0.02 μM, *p*<0.05, *n* =18; Figure S22). This suggests that **4**‐induced breast CSC death is related to intracellular ROS elevation. Having established that **4** is able to generate ROS in breast CSCs and that this trait contributes to its mechanism of toxicity, fluorescence microscopy studies were carried to determine if **4** could produce ROS in the ER of breast CSCs. HMLER‐shEcad cells treated with **4** (5 μM for 1 h at 37 °C), followed by ER‐Tracker Red (1.6 μM for 15 min) and the green‐emitting ROS indicator, DCFDA (20 μM for 10 min) revealed a high level of overlap between regions with elevated ROS and the ER (Figure S23). As expected, untreated cells did not display elevated ROS levels (Figure S24). This implies that **4** can indeed produce ROS in the ER of breast CSCs, which is a prerequisite for type II ICD inducers.

**Figure 4:**
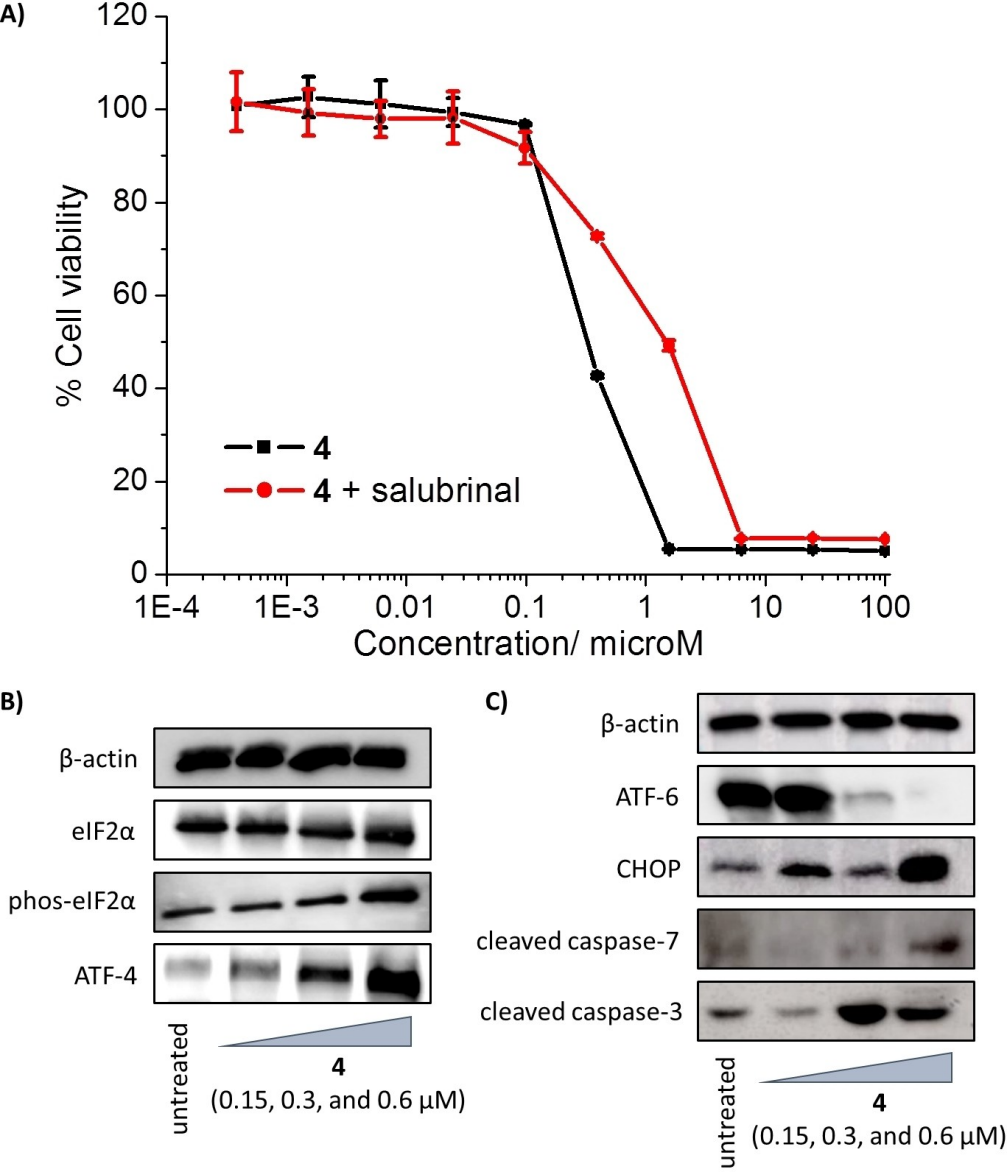
A) Representative dose–response curves for the treatment of HMLER‐shEcad cells with **4** after 72 h of incubation in the absence and presence of salubrinal (10 μM). B) Immunoblotting analysis of proteins related to the UPR. Protein expression in HMLER‐shEcad cells following treatment with **4** (0.15–0.6 μM for 2 h). C) Immunoblotting analysis of proteins related to the UPR and apoptosis. Protein expression in HMLER‐shEcad cells following treatment with **4** (0.15–0.6 μM for 72 h).

Next we investigated the possibility that **4** could induce ER stress in breast CSCs. Co‐administration of **4** and salubrinal (10 μM), a well‐known ER stress inhibitor,^[25]^ significantly reduced the cytotoxicity of **4** in HMLER‐shEcad cells. The IC_50_ value increased 4.7‐fold (1.49±0.01 μM) compared to that obtained from treatment with **4** alone (Figure 4A), suggesting that ER stress is a component of the cytotoxic mechanism of **4**. To further validate **4**‐mediated ER stress in breast CSCs, we monitored the expression of proteins related to the unfolded protein response (UPR).^[26]^ Upon incubation of HMLER‐shEcad cells with **4** (0.15–0.6 μM for 2 h), the expression of phosphorylated eukaryotic initiation factor 2α (phos‐eIF2α) increased while the expression of unphosphorylated eIF2α remained largely unaltered (Figure 4B). High phos‐eIF2α levels are known to promote selective translation of the stress‐related activating transcription factor‐4 (ATF‐4), which in turn can instigate apoptosis by upregulating C/EBP homologous protein (CHOP) expression.^[27]^ HMLER‐shEcad cells dosed with **4** (0.15–0.6 μM for 2 h) displayed higher levels of ATF‐4 compared to untreated cells (Figure 4B). Upon prolonged exposure of HMLER‐shEcad cells to **4** (0.15–0.6 μM for 72 h) the expression of the activating transcription factor‐6 (ATF‐6) dramatically decreased, consistent with its cleavage in response to ER stress (Figure 4C). Cleaved ATF‐6 can translocate to the nucleus and activate transcription of ER chaperones and (akin to ATF‐4) components of ER‐associated degradation such as CHOP.^[28]^ Indeed, CHOP was markedly upregulated in HMLER‐shEcad cells treated with **4** (0.15–0.60 μM for 72 h; Figure 4C). Similarly to **4**, HMLER‐shEcad cells treated with thapsigargin (300 nM for 1 h), a *bona fide* ER stress inducer, displayed higher expression levels of phos‐eIF2α, ATF‐4, and CHOP, and lower expression levels of ATF‐6 compared to untreated cells (Figure S25). Unrepaired ER stress and persistent activation of the UPR can lead to apoptosis.^[29]^ HMLER‐shEcad cells treated with **4** (0.15–0.60 μM for 72 h) displayed higher levels of cleaved caspase 3 and 7 compared to untreated cells (Figure 4C), characteristic of caspase‐dependent apoptosis. Taken together the immunoblotting and cytotoxicity studies show that **4** can induce ER stress which ultimately leads to apoptotic breast CSC death.

**Figure 5:**
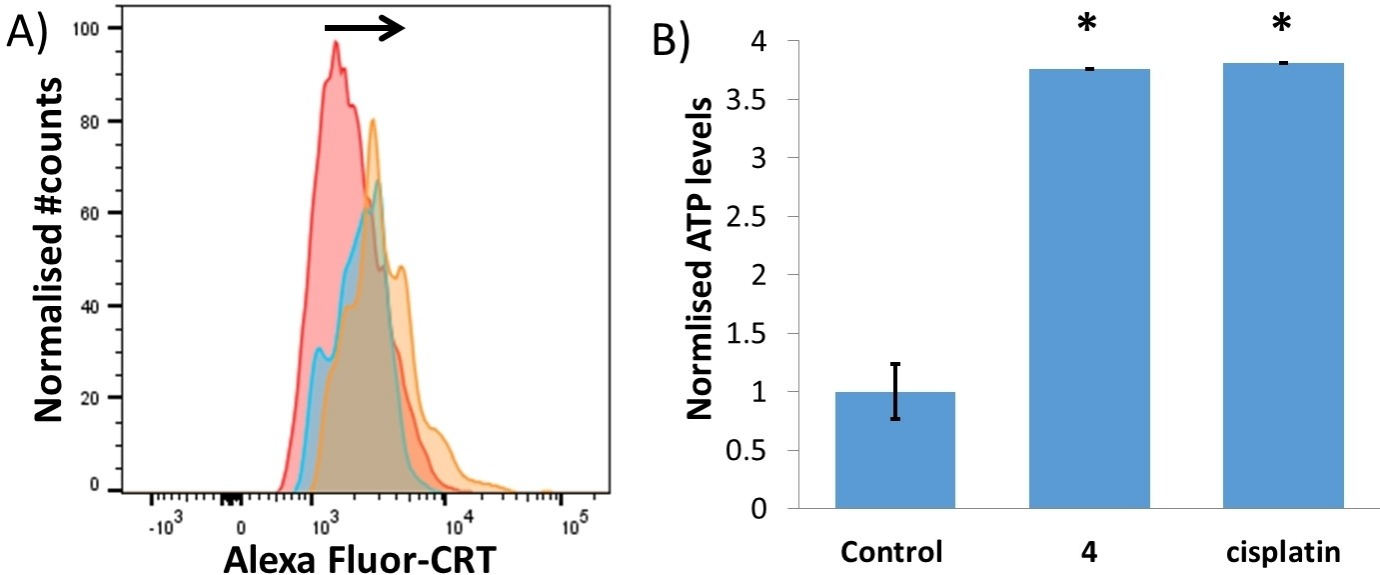
A) Representative histograms displaying the green fluorescence emitted by anti‐CRT Alexa Fluor 488 nm antibody‐stained untreated HMLER‐shEcad cells (red) and cells treated with **4** (IC_50_ value for 12 h; blue) or cisplatin (150 μM for 12 h) with thapsigargin (7 μM for 12 h; orange). B) Normalised extracellular ATP released from untreated HMLER‐shEcad cells and cells treated with **4** (IC_50_ value for 24 h) or cisplatin (IC_50_ value for 24 h). Error bars=SD and Student t‐test, * *p*<0.05.

ICD is characterised by the release or translocation of three major damage‐associated molecular patterns (DAMPs) namely, adenosine triphosphate (ATP), high mobility group box 1 (HMGB‐1), and calreticulin (CRT).^[8a]^ DAMPs are crucial to facilitating the phagocytic engulfment of apoptotic cells by immune cells. The presence of DAMPs was evaluated in breast CSC‐enriched HMLER‐shEcad cells treated with **4**. The translocation of CRT from the ER to the cell surface during early apoptosis is an early hallmark of ICD.^[8a]^ CRT acts as an “eat me” signal which promotes the phagocytosis of dying cells by immune cells.^[30]^ According to flow cytometric studies, HMLER‐shEcad cells treated with **4** (IC_50_ value for 12 h) displayed noticeably higher levels of CRT on their cell surface than untreated control cells (Figure 5A). As expected, HMLER‐shEcad cells co‐dosed with cisplatin (150 μM for 12 h) and thapsigargin (7 μM for 12 h; positive control) displayed similar levels of CRT cell surface exposure (Figure 5A). The majority of chemotherapeutic agents fail to induce ICD because they are unable to elicit CRT cell surface exposure even if they display other DAMPs.^[31]^ Therefore, the exposure of CRT by **4** is very promising in terms of ICD induction. ATP secreted from dying cells during the blebbing phase of apoptosis acts as a “find me” signal for immune cells.^[7]^ ATP secretion from HMLER‐shEcad cells treated with **4** (IC_50_ value for 24 h) and cisplatin (IC_50_ value for 24 h, positive control) was determined by analysing the supernatant using a luciferase‐based assay (Figure 5B). As depicted in Figure 5B, **4** induced a fourfold increase in extracellular ATP compared to untreated control cells, supporting the occurrence of ICD. As expected, cisplatin treatment also prompted significant (*p* <0.05, fourfold) ATP release (Figure 5B). The release of nuclear HMGB‐1 upon plasma membrane permeabilisation, serves as a cytokine and mediates ICD by promoting antigen processing and presentation (to T‐cells).^[32]^ The relative amount of HMGB‐1 in HMLER‐shEcad cells treated with **4** was assessed by immunoblotting studies to gauge potential HMGB‐1 release. HMLER‐shEcad cells treated with **4** (0.3 and 0.6 μM for 48 h) displayed markedly lower or undetectable amounts of HMGB‐1 relative to untreated control cells, indicative of HMGB‐1 expulsion (Figure S26). Intracellular HMGB‐1 was not detected in HMLER‐shEcad cells co‐treated with cisplatin (150 μM for 48 h) and thapsigargin (7 μM for 48 h; positive control; Figure S26), suggestive of HMGB‐1 release. Treatment with cisplatin (150 μM for 48 h) alone appeared to partially promote HMGB‐1 excretion (Figure S26). Taken together, the DAMP detection studies show that **4** induces CRT cell surface exposure, ATP release, and intracellular HMGB‐1 depletion in breast CSCs, and thus indicates that the **4**‐mediated breast CSC death profile is consistent with ICD.

**Figure 6:**
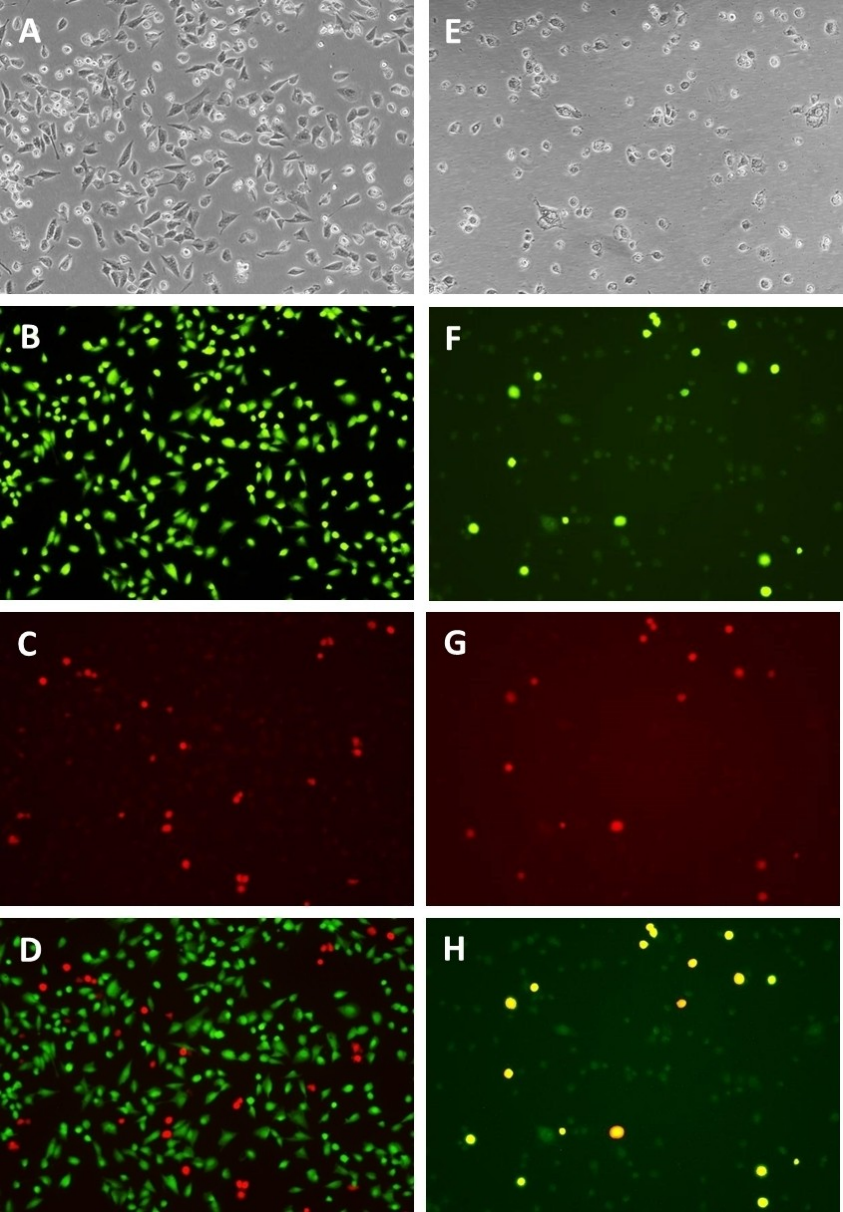
Fluorescence microscopy images (×10) of untreated CellTracker Green‐stained HMLER‐shEcad cells (left) or CellTracker Green‐stained HMLER‐shEcad cells treated with **4** (5 μM for 4 h; right), incubated with CellTracker Orange‐stained macrophages for 2 h. A), E) Bright‐field transmission image, B), F) green channel image, C), G) red channel image, and D), H) overlaid green and red channel images.

Having found that **4**‐treated breast CSC‐enriched HMLER‐shEcad cells displayed distinctive ICD features, we investigated the propensity of breast CSCs killed by **4** to undergo phagocytosis by macrophages using an *in vitro* assay. HMLER‐shEcad cells pre‐stained with CellTracker Green and incubated with **4** (5 μM for 4 h), were co‐treated with macrophages (obtained by differentiating acute monocytic leukaemia THP‐1 cells with phorbol 12‐myristate 13‐acetate, 100 nM for 72 h) prestained with CellTracker Orange, for 2 h. Phagocytosis, classified by the occurrence of double‐stained macrophages or CSCs, was monitored by fluorescence microscopy studies. The microscopy studies revealed a high level of overlap between **4**‐treated CellTracker Green‐stained HMLER‐shEcad cells and CellTracker Orange‐stained macrophages, indicative of effective phagocytosis (Figure 6A). Quantitative analysis of the images suggests that 97.8±3.1 % of CellTracker Orange‐stained macrophages overlapped with HMLER‐shEcad cells in the presence of **4**. Untreated CellTracker Green‐stained HMLER‐shEcad cells did not overlap with CellTracker Orange‐stained macrophages (0 % of CellTracker Orange‐stained macrophages overlapped with HMLER‐shEcad cells; Figure 6B). CellTracker Green‐stained HMLER‐shEcad cells dosed with cisplatin (50 μM for 4 h) or carboplatin (100 μM for 4 h) did not show significant phagocytosis by macrophages (4.6±1.0 % and 0 % of CellTracker Orange‐stained macrophages overlapped with HMLER‐shEcad cells, respectively; Figures S27 and S28). Collectively, this shows that **4** can kill breast CSCs in a manner that promotes phagocytosis by macrophages. Therefore, the copper(II) complex, **4** presented in this study has the potential to act as a type II ICD inducer in breast CSCs.

## 3 Conclusion

In summary, we show that a series of copper(II) complexes, **1**–**4** consisting of a Schiff base ligand and various polypyridyl ligands exhibit sub‐micromolar or low‐micromolar potency towards bulk breast cancer cells and breast CSCs. The most effective complex, **4** (bearing 4,7‐diphenyl‐1,10‐phenanthroline) killed breast CSCs 13 and 18 times better than salinomycin and cisplatin, respectively, in monolayer cell culture systems. Extraordinarily, **4** inhibited breast CSC‐enriched HMLER‐shEcad spheroids formation and viability favourably over nontumorigenic breast MCF10 A spheroids (> threefold selectivity), implying that **4** can potentially remove breast CSCs with reduced toxicity towards normal breast epithelial cells. In contrast, salinomycin killed HMLER‐shEcad spheroids and MCF10 A spheroids equipotently. Furthermore, **4** killed HMLER‐shEcad spheroids 34 and 25 times better than salinomycin and cisplatin, respectively. Detailed mechanistic studies revealed that **4** displayed all the common hallmarks of a Type II ICD inducer. For instance, **4** was able to enter the ER of breast CSCs and elevate ROS levels, leading to oxidative ER stress‐induced apoptosis. The ability of **4** to localise in the ER is likely to be due to the presence of the 4,7‐diphenyl‐1,10‐phenanthroline ligand, which is a common feature in many previously reported ER‐targeting or ER‐stress inducing metal complexes. Breast CSCs treated with **4** also displayed DAMPs such as CRT cell surface exposure, ATP release, and reduced intracellular HMGB‐1 expression, confirming that **4**‐mediated breast CSC death is consistent with ICD. Phagocytosis studies showed that breast CSCs dosed with **4** were effectively engulfed by macrophages *in vitro*. Phagocytosis is a critical, initial step in the immune response against bulk cancer cells and CSCs, and therefore this result highlights the promising immunogenic potential of **4**. To the best of our knowledge, **4** is the first metal complex to display both cytotoxic and immunogenic effects towards breast CSCs *in vitro*. Overall, our findings reinforce the therapeutic potential of copper‐containing compounds, and more specifically, provides the basis for their development as type II ICD inducers for CSC‐focused chemotherapy.

## Conflict of interest

The authors declare no conflict of interest.

## Supporting information

As a service to our authors and readers, this journal provides supporting information supplied by the authors. Such materials are peer reviewed and may be re‐organized for online delivery, but are not copy‐edited or typeset. Technical support issues arising from supporting information (other than missing files) should be addressed to the authors.

Supplementary
