# Supplementary material for: Immunogenic Cell Death of Breast Cancer Stem Cells Induced by an Endoplasmic Reticulum‐Targeting Copper(II) Complex
Source: Chembiochem. 2020 Sep 22;21(24):3618–24. doi: 10.1002/cbic.202000553 (PMC7757018; doi:10.1002/cbic.202000553)
Supplement: Supplementary file 1 — Supplementary [file CBIC-21-3618-s001.pdf]

ChemBioChem

Supporting Information

Immunogenic Cell Death of Breast Cancer Stem Cells Induced by an Endoplasmic Reticulum-Targeting Copper(II) Complex

Pooja Kaur, Alice Johnson, Joshua Northcote-Smith, Chunxin Lu, and Kogularamanan Suntharalingam*

Table of Content

Experimental Details

Figure S1. Chemical structures of the Schiff base ligand, $\mathbf{L^1}$ and the copper(II) complex, [Cu($\mathbf{L^1}$)Cl].

Figure S2. High resolution ESI mass spectrum (positive mode) of **1**.

Figure S3. High resolution ESI mass spectrum (positive mode) of **2**.

Figure S4. High resolution ESI mass spectrum (positive mode) of **3**.

Figure S5. High resolution ESI mass spectrum (positive mode) of **4**.

Figure S6. IR spectrum of (A) **1**, (B) **2**, (C) **3**, (D) **4**, (E) $\mathbf{L^1}$, and (F) [Cu($\mathbf{L^1}$)Cl] in the solid form.

Table S1. Crystallographic data for **3**.

Table S2. Selected bond lengths (Å) and angles (°) for **3**.

Table S3. Experimentally determined LogP values for **1-4**.

Figure S7. UV-Vis spectrum of **1** (25 μM) in PBS:DMSO (200:1) over the course of 24 h at 37 °C.

Figure S8. UV-Vis spectrum of **1** (25 μM) in mammary epithelial cell growth medium (MEGM):DMSO (200:1) over the course of 24 h at 37 °C.

Figure S9. Representative dose-response curves for the treatment of HMLER and HMLER-shEcad cells with **1** after 72 h incubation.

Figure S10. Representative dose-response curves for the treatment of HMLER and HMLER-shEcad cells with **2** after 72 h incubation.

Figure S11. Representative dose-response curves for the treatment of HMLER and HMLER-shEcad cells with **3** after 72 h incubation.

Figure S12. Representative dose-response curves for the treatment of HMLER and HMLER-shEcad cells with **4** after 72 h incubation.

Figure S13. Representative dose-response curves for the treatment of HMLER and HMLER-shEcad cells with [Cu($\mathbf{L}^1$)Cl] after 72 h incubation.

Figure S14. Quantification of spheroid formation with HMLER-shEcad cells untreated and treated with **1-4**, [Cu($\mathbf{L}^1$)Cl], or salinomycin at their respective IC_{20} values for 5 days. Error bars = SD and Student t-test, * = $p < 0.05$.

Figure S15. Representative bright-field images (× 20) of HMLER-shEcad spheroids in the absence and presence of **1-4** or salinomycin at their respective IC_{20} values for 5 days.

Figure S16. Representative bright-field images (× 20) of HMLER-shEcad spheroids in the absence and presence of [Cu($\mathbf{L}^1$)Cl] at the respective IC_{20} values for 5 days.

Figure S17. Representative dose-response curves for the treatment of HMLER-shEcad spheroids with **1-4** and [Cu($\mathbf{L}^1$)Cl].

Figure S18. Representative dose-response curve for the treatment of MCF10A spheroids with **4**.

Figure S19. Representative dose-response curve for the treatment of MCF10A spheroids with salinomycin.

Figure S20. Copper content in whole cell and endoplasmic reticulum fractions isolated from HMLER-shEcad cells treated with **4** or [Cu($\mathbf{L}^1$)Cl] (5 μM for 24 h at 37°C). Error bars = SD.

Figure S21. Normalised ROS activity in untreated HMLER-shEcad cells (control) and HMLER-shEcad cells treated with **4** (IC_{50} value for 1, 3, 6, 16, 24, and 48 h). Error bars = SD and Student t-test, * = $p < 0.05$.

Figure S22. Representative dose-response curves for the treatment of HMLER-shEcad cells with **4** after 72 incubation in the presence of *N*-acetylcysteine (2.5 mM).

Figure S23. Fluorescence microscopy images (× 20) of live HMLER-shEcad cells co-treated with **4** (5 μM for 1 h), ER-Tracker Red (1.6 μM for 15 min), and green-emitting DCFDA (20 μM for 10 min). (A) Bright field transmission image (B) green channel image (C) red channel image, and (D) overlaid bright field transmission, green channel, and red channel image.

Figure S24. Fluorescence microscopy images (× 20) of live HMLER-shEcad cells co-treated with ER-Tracker Red (1.6 μM for 15 min) and green-emitting DCFDA (20 μM for 10 min). (A) Bright field transmission image (B) green channel image (C) red channel image, and (D) overlaid bright field transmission, green channel, and red channel image.

Figure S25. Immunoblotting analysis of proteins related to the unfolded protein response (UPR). Protein expression in HMLER-shEcad cells following treatment with thapsigargin (300 nM for 1 h).

Figure S26. Immunoblotting analysis of high mobility group box 1 (HMGB-1). Protein expression in HMLER-shEcad cells following treatment with **4** (0.3 and 0.6 μM for 48 h) or cisplatin (150 μM for 48 h) and thapsigargin (7 μM for 48 h) or, cisplatin (150 μM for 48 h).

Figure S27. Fluorescence microscopy images (× 10) of CellTracker Green-stained HMLER-shEcad cells treated with cisplatin (50 μM for 4 h), and then incubated with CellTracker Orange-stained macrophages for 2 h. (A) Bright field transmission image, (B) green channel image, (C) red channel image, and (D) overlaid green channel and red channel images.

Figure S28. Fluorescence microscopy images (× 10) of CellTracker Green-stained HMLER-shEcad cells treated with carboplatin (100 μM for 4 h), and then incubated with CellTracker Orange-stained macrophages for 2 h. (A) Bright field transmission image, (B) green channel image, (C) red channel image, and (D) overlaid green channel and red channel images.

References

Experimental Details

Materials and Methods. All synthetic procedures were performed under normal atmospheric conditions or under nitrogen. High-resolution electron spray ionisation mass spectra were obtained by Dr Lisa Haigh (Imperial College London) on a Bruker Daltonics Esquire 3000 spectrometer. Fourier transform infrared (FTIR) spectra were obtained with an IR Affinity-1S Shimadzu spectrophotometer. Elemental analysis was performed commercially by London Metropolitan University. For all cellular studies, 10 mM stock solutions were prepared in dimethyl sulfoxide and then diluted to the appropriate concentration using biological solutions. 1,10-Phenanthroline, 5-methyl-1,10-phenanthroline, 3,4,7,8-Tetramethyl-1,10-phenanthroline, and 4,7-diphenyl-1,10-phenanthroline were purchased from Sigma and used as received. The Schiff base ligand, $\mathbf{L^1}$ (2-(methylthio)ethylsalicylaldimine) was prepared according to previously reported protocols.[1]

Synthesis [Cu^{II}(L^1)(1,10-phenanthroline)], 1. To $Cu(NO_3)_2$•$3H_2O$ (110.3 mg, 0.46 mmol) dissolved in methanol (10 mL), 1,10-phenanthroline (87.4 mg, 0.48 mmol) was added slowly and stirred at room temperature for 30 min. $\mathbf{L^1}$ (89.9 mg, 0.46 mmol) in methanol (10 mL) was then added dropwise, and the dark green reaction mixture was stirred for 72 h at room temperature. The reaction mixture was filtered to remove unreacted material and $NaPF_6$ (77.3 mg, 0.46 mmol) in methanol (10 mL) was added to the filtrate and stirred at room temperature for 30 min. The resulting precipitate was filtered, washed thoroughly with cold methanol (10 mL) and diethyl ether (8 mL), and dried to obtain **1** as a light green solid (31.9 mg, 12%); IR (solid, cm^{-1}): 3088, 1612, 1591, 1524, 1430, 1292, 1145, 1107, 1027, 879, 833, 777, 719, 649, 556; HRMS (ESI) (DMSO) Calcd. for $C_{23}H_{23}CuN_3O_2S$ $[M-PF_6-H+CH_3OH]^+$: 468.6971, Found: $[M-PF_6-H+CH_3OH]^+$: 468.0421; EA Anal. Calcd. for **1** ($C_{22}H_{20}CuF_6N_3OPS$): C, 45.33; H, 3.46; N, 7.21. Found: C, 45.22; H, 3.19; N, 7.50.

Synthesis of [Cu^{II}(L^1)(5-methyl-1,10-phenanthroline)], 2. To $Cu(NO_3)_2$•$3H_2O$ (126.4 mg, 0.52 mmol) dissolved in methanol (8 mL), 5-methyl-1,10-phenanthroline (106.3 mg, 0.55 mmol) in methanol (5 mL) was added and stirred at room temperature for 30 min. $\mathbf{L^1}$ (99.2 mg, 0.51 mmol) in methanol (5 mL) was then added slowly, and the dark blue reaction mixture was stirred overnight at room temperature. $NaPF_6$ (90.3 mg, 0.54 mmol) was added, and the reaction mixture was stirred for 30 minutes. The resulting precipitate was filtered, washed thoroughly with cold methanol (10 mL) and diethyl ether (8 mL), and dried to obtain **2** as a pale green-blue solid (126.3 mg, 41%); IR (solid, cm^{-1}): 3088, 1620, 1608, 1592, 1526, 1490, 1428, 1288, 1227, 1157, 1120, 1075, 1022, 878, 833, 800, 726, 665, 649, 620, 554, 423, 403; HRMS (ESI) (DMSO) Calcd. for $C_{23}H_{22}CuN_3OS$ $[M-PF_6]^+$: 451.6943, Found: $[M-PF_6]^+$: 451.0773; EA Anal. Calcd. for **2** ($C_{23}H_{22}CuF_6N_3OPS$): C, 46.27; H, 3.71; N, 7.04. Found: C, 46.42; H, 3.58; N, 7.30

Synthesis of [Cu^{II}(L^1)(3,4,7,8-tetramethyl-1,10-phenanthroline)], 3. $Cu(CH_3COO)_2$•H_2O (59.9 mg, 0.30 mmol) and 3,4,7,8-tetramethyl-1,10-phenanthroline (70.9 mg, 0.30 mmol) were dissolved in methanol (5 mL) and stirred at room temperature for 30 min. $\mathbf{L^1}$ (58.6 mg, 0.30 mmol) in methanol (5 mL) was then added and the reaction mixture was stirred for 20 h at room temperature. $NaPF_6$ (50.4 mg, 0.30 mmol) was then added, and the reaction mixture was stirred for 30 minutes. The resulting precipitate was filtered, washed thoroughly with cold methanol (10 mL) and diethyl ether (8 mL), and dried to obtain **3** as a dark green solid (130.0 mg, 68%); IR (solid, cm^{-1}): 2919, 1619, 1600, 1537, 1525, 1467, 1447, 1428, 1396, 1322, 1198, 1147, 1127, 1033, 822, 763, 740, 724, 653, 642, 618, 599, 556, 520, 454, 422, 403; HRMS (ESI) (DMSO) Calcd. for $C_{26}H_{28}CuN_3OS$ $[M-PF_6]^+$: 493.7412, Found: [M-

$PF_6]^+$: 493.1253; EA Anal. Calcd. for **3** ($C_{26}H_{28}CuF_6N_3OPS$)•$1.5H_2O$: C, 46.88; H, 4.69; N, 6.31. Found: C, 46.98; H, 4.47; N, 6.15.

Synthesis of [Cu^{II}(L^1)(4,7-diphenyl-1,10-phenanthroline)], 4. $Cu(NO_3)_2$•$3H_2O$ (46.2 mg, 0.19 mmol) and 4,7-diphenyl-1,10-phenanthroline (63.1 mg, 0.19 mmol) were dissolved in methanol (4 mL) and the resultant blue solution was stirred for 30 min at room temperature. **L^1** (35.7 mg, 0.18 mmol) in methanol (10 mL) was then added dropwise, and the dark green reaction mixture was stirred for 72 h at room temperature. A solution of $NaPF_6$ (35.1 mg, 0.21 mmol) in methanol (5 mL) was added, the reaction mixture was stirred at room temperature for 30 min. The resulting precipitate was filtered, washed thoroughly with cold methanol (10 mL) and diethyl ether (8 mL), and dried to obtain **4** as a light green solid (33.8 mg, 24%); IR (solid, cm^{-1}): 3084, 1620, 1604, 1560, 1523, 1495, 1426, 1402, 1285, 1236, 1194, 1091, 1026, 1002, 826, 766, 737, 701, 669, 636, 556, 544, 491, 475, 455, 418, 406; HRMS (ESI) (DMSO) Calcd. for $C_{34}H_{28}CuF_6N_3OS$ $[M-PF_6]^+$: 589.7411, Found: $[M-PF_6]^+$: 589.1256; EA Anal. Calcd. for **4** ($C_{34}H_{28}CuF_6N_3OPS$): C, 55.55; H, 3.84; N, 5.72. Found: C, 55.78; H, 3.58; N, 5.62.

Synthesis of [Cu(L^1)Cl]. L^1 (108.8 mg, 0.56 mmol) in methanol (5 mL) was neutralised with triethylamine (approx. 4 drops) and stirred for 5 min. $CuCl_2$ (76.9 mg, 0.57 mmol) in methanol (2 mL) was added slowly, and the dark green solution was stirred overnight. The resulting precipitate was filtered to yield [Cu(**L^1**)Cl] as a light green solid (60.3 mg, 36%); IR (solid, cm^{-1}): 2982, 2924, 1627, 1597, 1538, 1472, 1450, 1402, 1311, 1197, 1153, 1127, 1036, 979, 900, 795, 760, 603, 534, 512, 451; HRMS (ESI) (DMSO): Calcd. for $C_{12}H_{18}CuNO_2S_2$ $[M-Cl+DMSO]^+$: 335.6244, Found: $[M-Cl+DMSO]^+$: 355.0086; EA: Anal. Calcd. for [Cu(**L^1**)Cl] ($C_{10}H_{12}ClCuNOS$): C, 40.96; H, 4.12; N, 4.78. Found: C, 40.62; H, 4.31; N, 4.82.

X-ray Single Crystal Diffraction Analysis. Standard procedures were used to mount the crystal on a Gemini diffractometer with graphite-monochromated Cu Kα radiation (λ = 1.54178 Å) at 293 K. The crystal structure was solved using direct methods in SHELXS and refined by full-matrix least-squares routines, based on F^2, using the SHELXL program.[2] All the H atoms were placed in geometrically idealised positions and constrained to ride on their parent atoms. The structure has been deposited with the Cambridge Crystallographic Data Centre (CCDC 1943504). This information can be obtained free of charge from www.ccdc.cam.ac.uk/data_request/cif.

Measurement of water-octanol partition coefficient (LogP). The LogP value for **1-4** was determined using the shake-flask method. The octanol used in this experiment was pre-saturated with water. An aqueous solution of **1-4** (500 μL, 100 μM) was incubated with octanol (500 μL) in a 1.5 mL tube. The tube was shaken at room temperature for 24 h. The two phases were separated by centrifugation and the **1-4** content in the aqueous phase was determined by inductively coupled plasma mass spectrometry (ICP-MS, PerkinElmer NexION 350D).

Cell Lines and Cell Culture Conditions. The human mammary epithelial cell lines, HMLER and HMLER-shEcad were kindly donated by Prof. R. A. Weinberg (Whitehead Institute, MIT). The human epithelial breast MCF710A and acute monocytic leukemia THP-1 cell lines were acquired from the American Type Culture Collection (ATCC, Manassas, VA, USA). HMLER, HMLER-shEcad, and MCF10A cells were maintained in Mammary Epithelial Cell Growth Medium (MEGM) with supplements and growth factors (BPE,

hydrocortisone, hEGF, insulin, and gentamicin/amphotericin-B). THP-1 cells were maintained in RPMI 1640 medium with 2 mM L-glutamine, 10% FBS. The cells were grown at 310 K in a humidified atmosphere containing 5% CO_2.

Cytotoxicity MTT assay. The colorimetric MTT assay was used to determine the toxicity of **1-4** and [Cu($\mathbf{L^1}$)Cl]. HMLER and HMLER-shEcad cells (5×10^3) were seeded in each well of a 96-well plate. After incubating the cells overnight, various concentrations of the compounds (0.0004-100 μM), were added and incubated for 72 h (total volume 200 μL). Stock solutions of the compounds were prepared as 10 mM solutions in DMSO and diluted using media. The final concentration of DMSO in each well did not exceed 1% and this amount was present in the untreated control as well. After 72 h, 20 μL of a 4 mg/mL solution of MTT in PBS was added to each well, and the plate was incubated for an additional 4 h. The MEGM/MTT mixture was aspirated and 200 μL of DMSO was added to dissolve the resulting purple formazan crystals. The absorbance of the solutions in each well was read at 550 nm. Absorbance values were normalized to (DMSO-containing) control wells and plotted as concentration of test compound versus % cell viability. IC_{50} values were interpolated from the resulting dose dependent curves. The reported IC_{50} values are the average of three independent experiments, each consisting of six replicates per concentration level (overall n = 18).

Spheroid Formation and Viability Assay. HMLER-shEcad and MCF10A cells (5×10^3) were plated in ultralow-attachment 96-well plates (Corning) and incubated in MEGM supplemented with B27 (Invitrogen), 20 ng/mL EGF, and 4 μg/mL heparin (Sigma) for 5 days. Studies were also conducted in the presence of **1-4**, [Cu($\mathbf{L^1}$)Cl], and salinomycin (0-133 μM). Spheroids treated with **1-4**, [Cu($\mathbf{L^1}$)Cl], and salinomycin (at their respective IC_{20} values, 5 days) were counted and imaged using an inverted microscope. The viability of the spheroids was determined by addition of a resazurin-based reagent, TOX8 (Sigma). After incubation for 16 h, the solutions were carefully transferred to a black 96-well plate (Corning), and the fluorescence of the solutions was read at 590 nm (λ_{ex} = 560 nm). Viable spheroids reduce the amount of the oxidized TOX8 form (blue) and concurrently increase the amount of the fluorescent TOX8 intermediate (red), indicating the degree of spheroid cytotoxicity caused by the test compound. Fluorescence values were normalized to DMSO-containing controls and plotted as concentration of test compound versus % spheroid viability. IC_{50} values were interpolated from the resulting dose dependent curves. The reported IC_{50} values are the average of three independent experiments, each consisting of three replicates per concentration level (overall n = 9).

Cellular Uptake. To measure the cellular uptake of **4** and [Cu($\mathbf{L^1}$)Cl] *ca.* 1 million HMLER-shEcad cells were treated with **4** and [Cu($\mathbf{L^1}$)Cl] (5 μM) at 37 °C for 24 h. After incubation, the media was removed and the cells were washed with PBS (2 mL × 3), and harvested. The number of cells was counted at this stage, using a haemocytometer. This mitigates any cell death induced by **4** and [Cu($\mathbf{L^1}$)Cl] at the administered concentration and experimental cell loss. The cells were centrifuged to form pellets. The cellular pellets were dissolved in 65% HNO_3 (250 μL) overnight. The cellular pellets were also used to determine the copper content in the endoplasmic reticulum (ER) fraction. The Sigma-Aldrich Endoplasmic Reticulum Isolation Kit was used to extract and separate the ER fraction. The fractions were dissolved in 65% HNO_3 overnight (250 μL final volume). All samples were diluted 5-fold with water and analysed using inductively coupled plasma mass spectrometry (ICP-MS, PerkinElmer NexION 350D). Copper levels are expressed as Cu (ppb) per million cells. Results are presented as the mean of five determinations for each data point.

Intracellular ROS Assay. HMLER-shEcad cells (5 × 10^3) were seeded in each well of a 96-well plate. After incubating the cells overnight, they were treated with **4** (IC_{50} value for 1-48 h) and incubated with 6-carboxy-2′,7′-dichlorodihydrofluorescein diacetate (20 μM) for 30 min. The intracellular ROS level was determined by measuring the fluorescence of the solutions in each well at 529 nm (λ_{ex} = 504 nm).

Fluorescence Microscopy. HMLER-shEcad cells (1×10^4) were incubated with **4** (5 μM for 1 h) (using phenol-free MEGM cell media). The media was then removed and the cells were washed with additional media (2 mL × 2). After incubation of the cells with more media containing ER-Tracker Red (1.6 μM for 15 min), the media was removed, the cells were washed with additional media (2 mL × 2). Then the cells were incubated with more media containing the green-emitting ROS indicator, DCFDA (20 μM for 10 min), the media was removed, the cells were washed with additional media (2 mL × 2), and imaged using a fluorescent microscope. Fluorescence imaging experiments were performed using a Nikon Eclipse Ts2-FL microscope. The microscope was operated with the NIS Element software. The exposure time for acquisition of fluorescence images was kept constant for each series of images at each channel. The images were overlaid using ImageJ (version 1.45, NIH).

Immunoblotting Analysis. HMLER-shEcad cells (5 × 10^3 cells) were incubated with **4** (0.15-0.6 μM for 2h or 72 h) or thapsigargin (300 nM for 1 h) at 37 ^{o}C. Cells were washed with PBS, scraped into SDS-PAGE loading buffer (64 mM Tris-HCl (pH 6.8)/ 9.6% glycerol/ 2%SDS/ 5% β-mercaptoethanol/ 0.01% Bromophenol Blue), and incubated at 95 ^{o}C for 10 min. Whole cell lysates were resolved by 4-20 % sodium dodecylsulphate polyacylamide gel electrophoresis (SDS-PAGE; 200 V for 25 min) followed by electro transfer to polyvinylidene difluoride membrane, PVDF (350 mA for 1 h). Membranes were blocked in 5% (w/v) non-fat milk in PBST (PBS/0.1% Tween 20) and incubated with the appropriate primary antibodies (Cell Signalling Technology). After incubation with horseradish peroxidase-conjugated secondary antibodies (Cell Signalling Technology), immune complexes were detected with the ECL detection reagent (BioRad) and analysed using a chemiluminescence imager (Bio-Rad ChemiDoc Imaging System).

CRT cell surface exposure. Flow cytometry was used to analyse cell surface CRT exposure. HMLER-shEcad cells were seeded into a 6-well plate (at a density of 5 × 10^5 cells/ mL) and the cells were incubated at 37°C overnight. The cells were treated with **4** (IC_{50} value) or co-treated with cisplatin (150 μM) with thapsigargin (7 μM) for 12 hours at 37°C. The cells were then harvested by trypsinization and collected by centrifugation. The pellet was suspended in PBS (500 μL), and after the addition of the Alexa Fluor® 488 nm labelled anti-CRT antibody (5 μl), the cells were incubated in the dark for 30 minutes. The cells were then washed with PBS (1 mL) and analysed using a FACSCanto II flow cytometer (BD Biosciences) (10,000 events per sample were acquired). The FL1 channel was used to assess CRT cell surface exposure. Cell populations were analysed using the FlowJo software (Tree Star).

ATP assay. HMLER-shEcad cells (5 × 10^3 cells /well) were seeded in a 96-well plate and incubated overnight. The cells were then treated with **4** (IC_{50} value) or cisplatin (IC_{50} value, positive control) for 24 hours at 37°C. The media was carefully extracted and transferred into a white-walled opaque 96-well plate, and a luciferin-based ENLITEN ATP Assay Kit (Promega) was used to measure the relative amount of ATP released into the supernatant.

HMGB-1 release. HMLER-shEcad cells (1 × 10^6 cells) were incubated with **4** (0.3 and 0.6 μM) or cisplatin (150 μM) for 48 h at 37°C to induce cell death. As a positive control, HMGB-1 release was induced by the co-treatment of cisplatin (150 μM) with thapsigargin (7 μM) for 48 h. Cells were collected in full and added to SDS-PAGE loading buffer (64 mM Tris-HCl (pH 6.8)/ 9.6% glycerol/ 2%SDS/ 5% β-mercaptoethanol/ 0.01% Bromophenol Blue) and incubated at 95°C for 10 min. The HMGB-1 content was probed by immunoblotting analysis as described above. The anti-HMGB-1 antibody (Cell Signalling Technology) was used in this experiment.

Phagocytosis assay. HMLER-shEcad cells were seeded into a 6-well plate (at a density of 5 × 10^5 cells/ mL) and the cells were incubated at 37°C overnight. The cells were stained with CellTracker Green (30 min) and washed with MEGM media. The cells were then treated with **4** (5 μM), cisplatin (50 μM), or carboplatin (100 μM) for 4 h at 37°C. Then macrophages, obtained by differentiating THP-1 cells with phorbol 12-myristate 13-acetate (100 nM for 72 h) and pre-stained with CellTracker Orange for 30 min and washed with RPMI 1640 media, were added to the HMLER-shEcad cells (at a density of 1 × 10^5 cells/ mL). After 2 h, phagocytosis was assessed by fluorescence imaging experiments using a Nikon Eclipse Ts2-FL microscope. The microscope was operated with the NIS Element software. The exposure time for acquisition of fluorescence images was kept constant for each series of images at each channel. The images were overlaid using ImageJ (version 1.45, NIH).

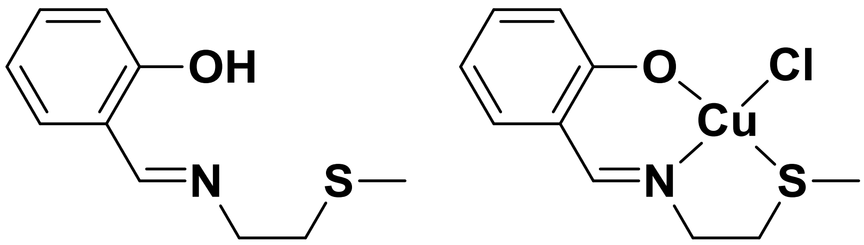

L^1 [Cu(**L**^{1})Cl]

Figure S1. Chemical structures of the Schiff base ligand, **L**^{1} and the copper(II) complex, [Cu(**L**^{1})Cl].

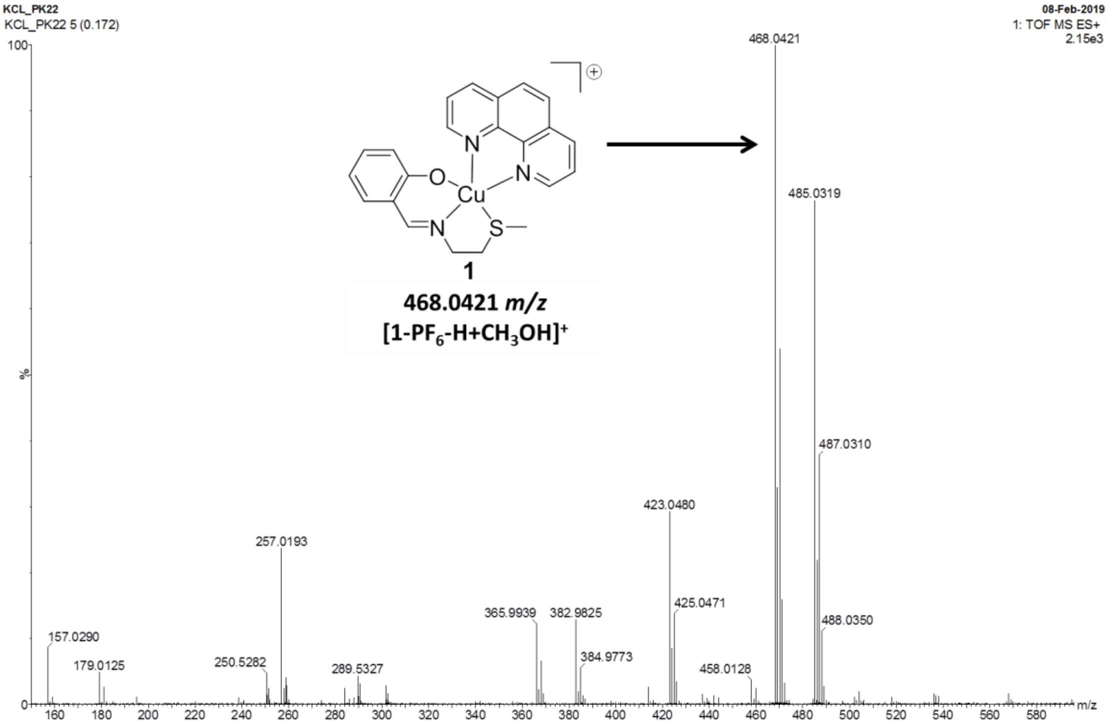

Figure S2. High resolution ESI mass spectrum (positive mode) of **1**.

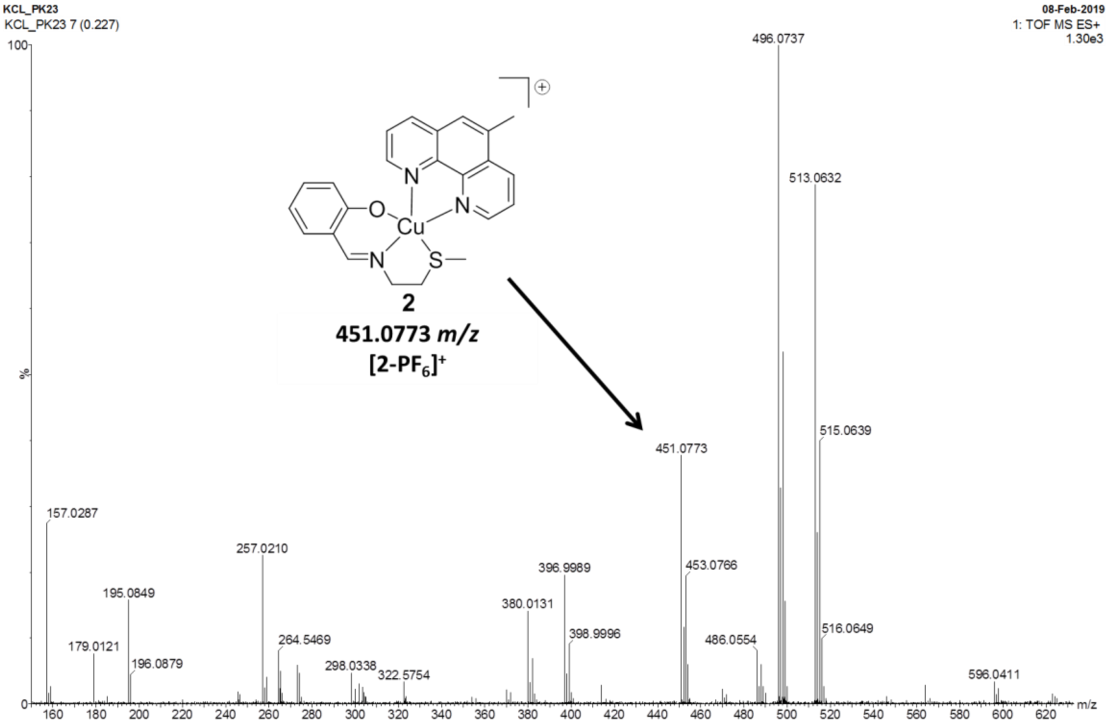

Figure S3. High resolution ESI mass spectrum (positive mode) of **2**.

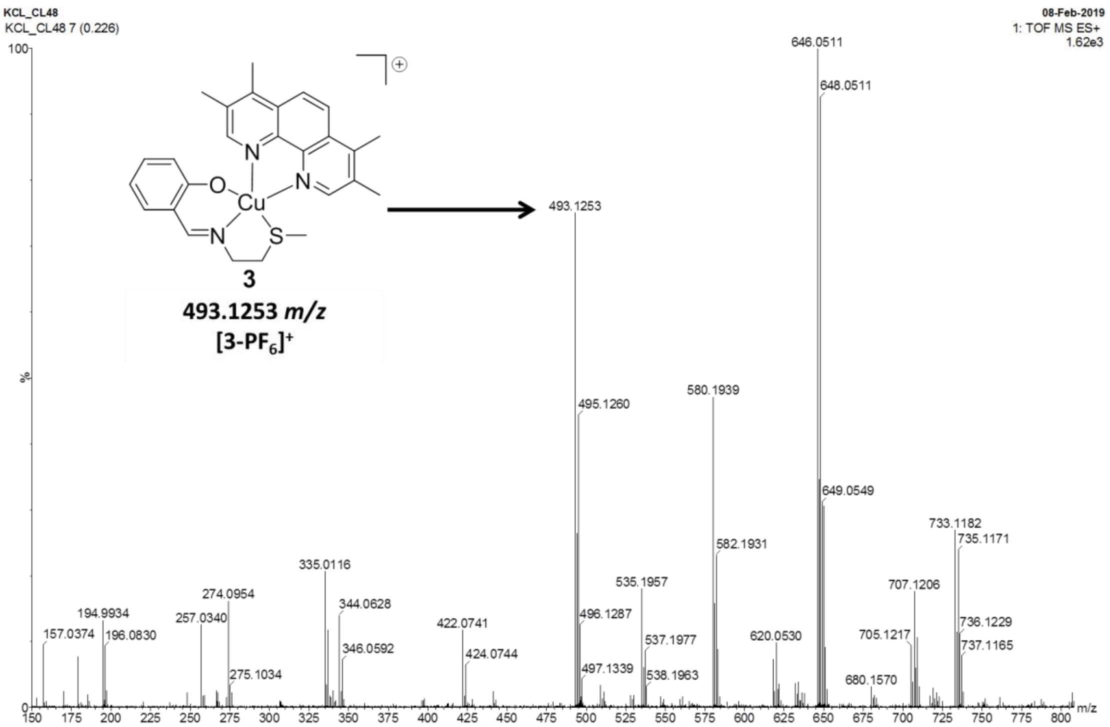

Figure S4. High resolution ESI mass spectrum (positive mode) of **3**.

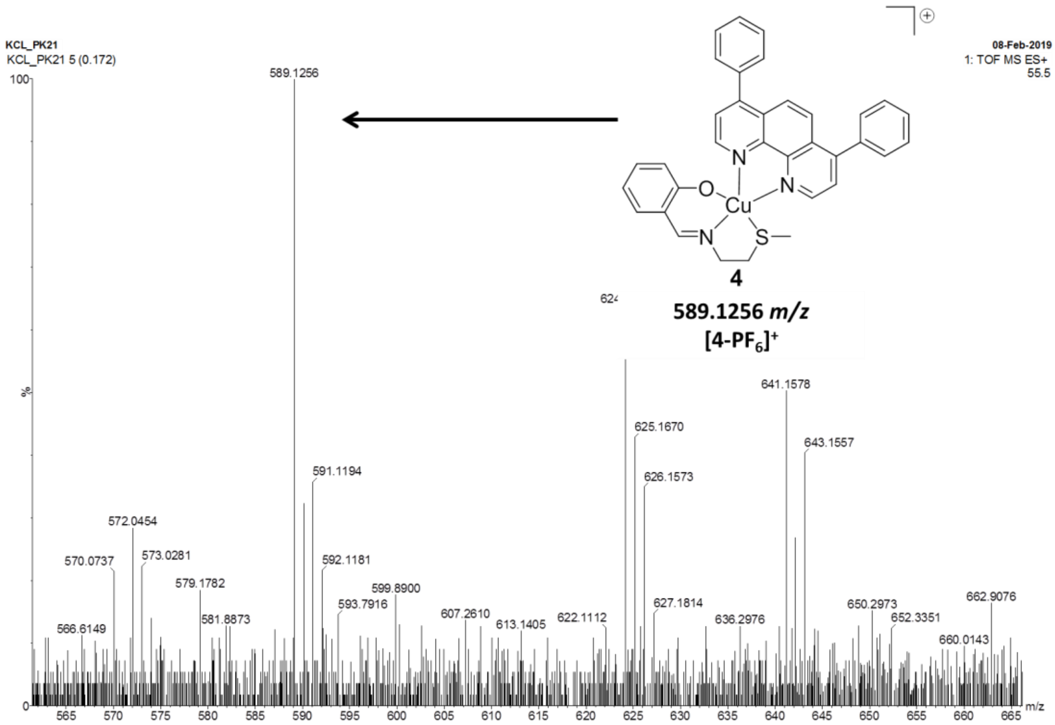

Figure S5. High resolution ESI mass spectrum (positive mode) of **4**.

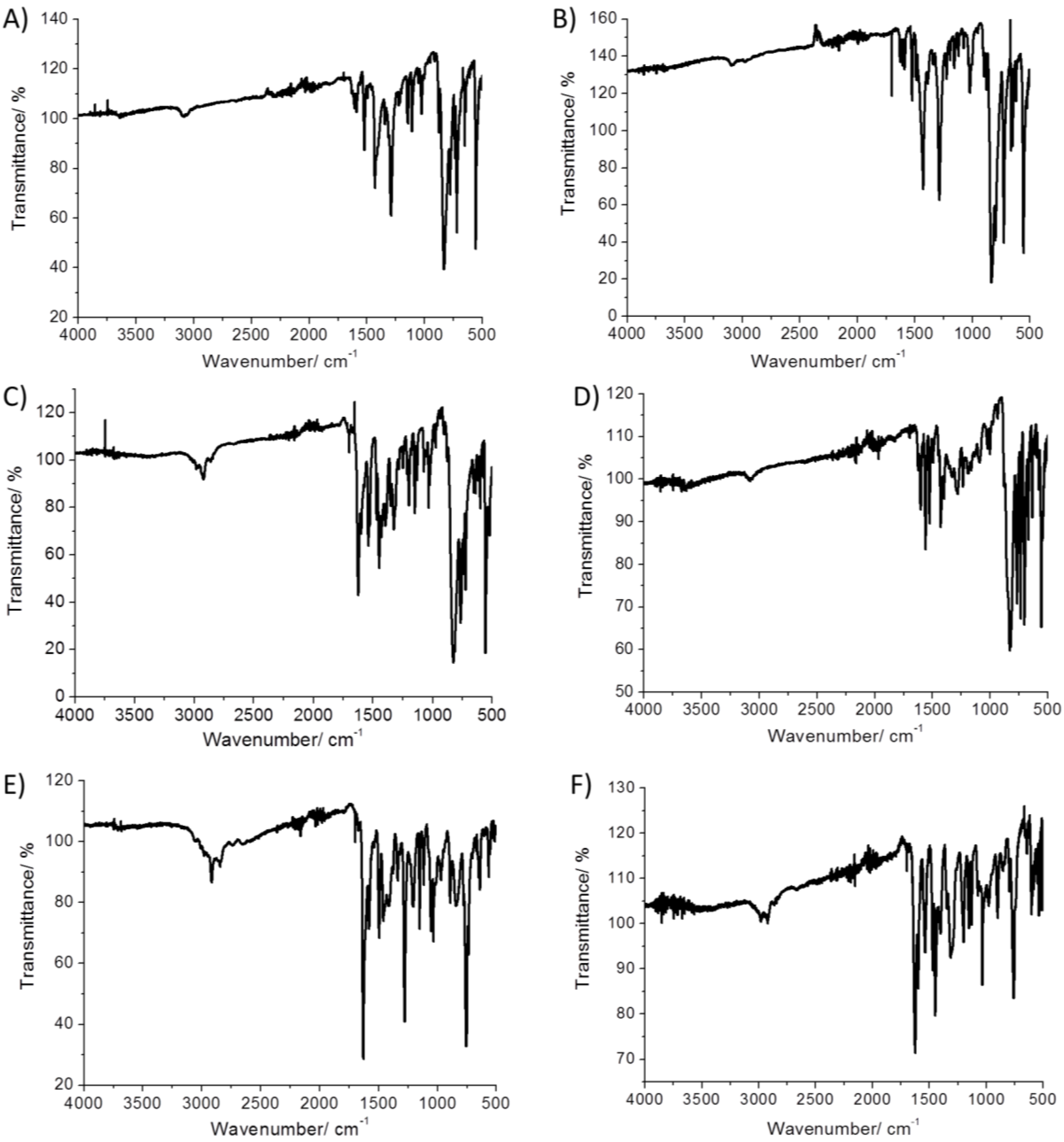

Figure S6. IR spectrum of (A) **1**, (B) **2**, (C) **3**, (D) **4**, (E) $\mathbf{L^1}$, and (F) [Cu($\mathbf{L^1}$)Cl] in the solid form.

Table S1. Crystallographic data for **3**.

formula	$C_{26}H_{28}CuF_6N_3OPS$
Fw	639.09
crystal system	monoclinic
space group	*P21/n*
a, Å	13.6437(2)
b, Å	13.7874(2)
c, Å	15.7658(3)
α, deg.	90
β, deg.	114.193(2)
γ, deg.	90
V, Å^3	2705.24(9)
Z	4
D_{calcd}, Mg/m^3	1.569
Reflections collected	10413
Reflections independent (R_{int})	5227 (0.0218)
Goodness-of-fit on F^2	1.049
$R(I > 2\sigma I)$	0.0380, 0.1052

Table S2. Selected bond lengths (Å) and angles (°) for **3**.

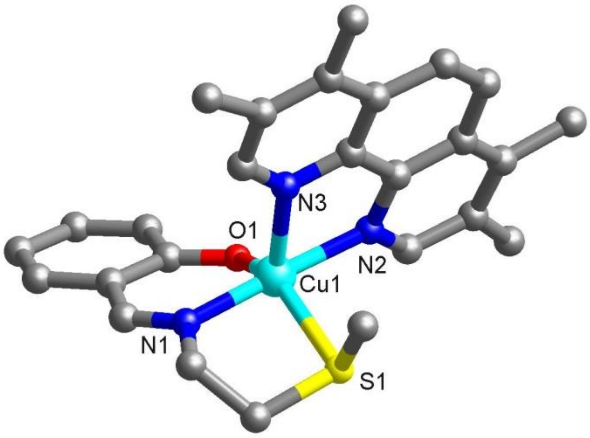

Cu(1)-O(1)	1.9122(16)	Cu(1)-N(1)	1.9408(18)
Cu(1)-N(2)	2.0017(17)	Cu(1)-N(3)	2.1797(18)
Cu(1)-S(1)	2.4589(7)	O(1)-Cu(1)-N(1)	93.91(7)
O(1)-Cu(1)-N(2)	88.61(7)	N(1)-Cu(1)-N(3)	100.89(8)
O(1)-Cu(1)-N(3)	120.29(7)	N(1)-Cu(1)-N(2)	177.29(8)
N(2)-Cu(1)-N(3)	78.62(7)	O(1)-Cu(1)-S(1)	141.93(6)
N(1)-Cu(1)-S(1)	85.21(6)	N(3)-Cu(1)-S(1)	97.08(5)
N(2)-Cu(1)-S(1)	92.20(5)		

Table S3. Experimentally determined LogP values for **1-4**.

Metal complex	LogP
**1**	0.73 ± 0.07
**2**	0.75 ± 0.07
**3**	1.15 ± 0.18
**4**	2.01 ± 0.16

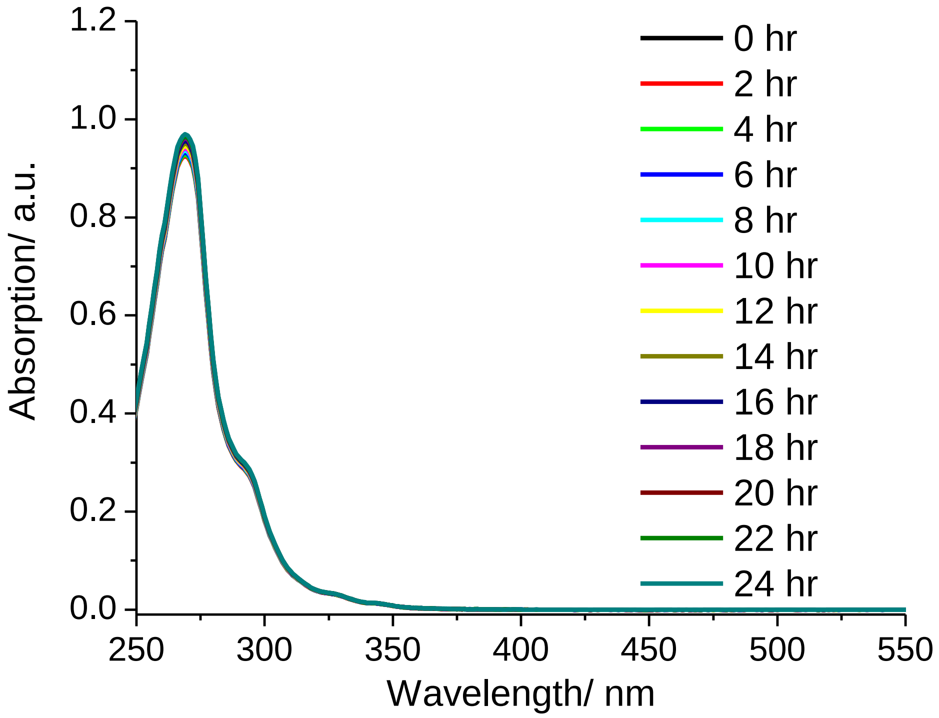

Figure S7. UV-Vis spectrum of **1** (25 µM) in PBS:DMSO (200:1) over the course of 24 h at 37 °C.

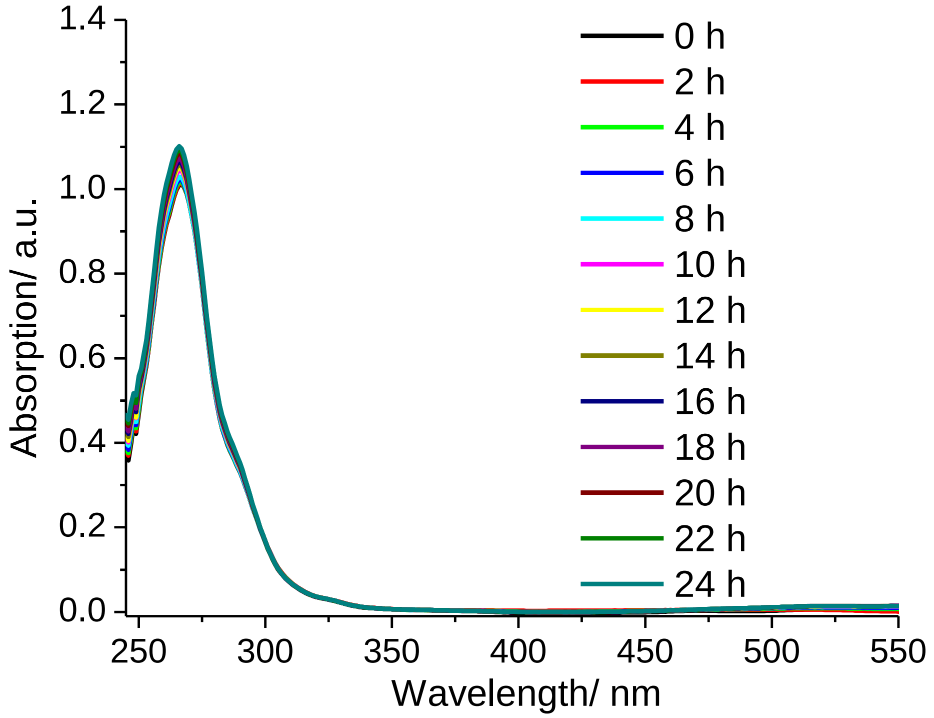

Figure S8. UV-Vis spectrum of **1** (25 µM) in mammary epithelial cell growth medium (MEGM):DMSO (200:1) over the course of 24 h at 37 °C.

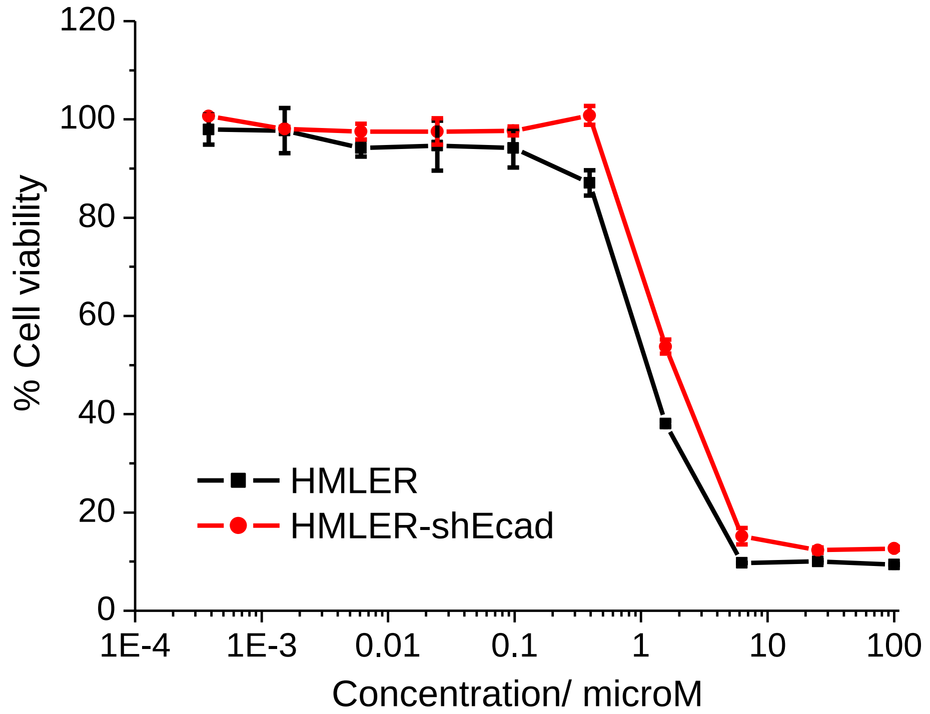

Figure S9. Representative dose-response curves for the treatment of HMLER and HMLER-shEcad cells with **1** after 72 h incubation.

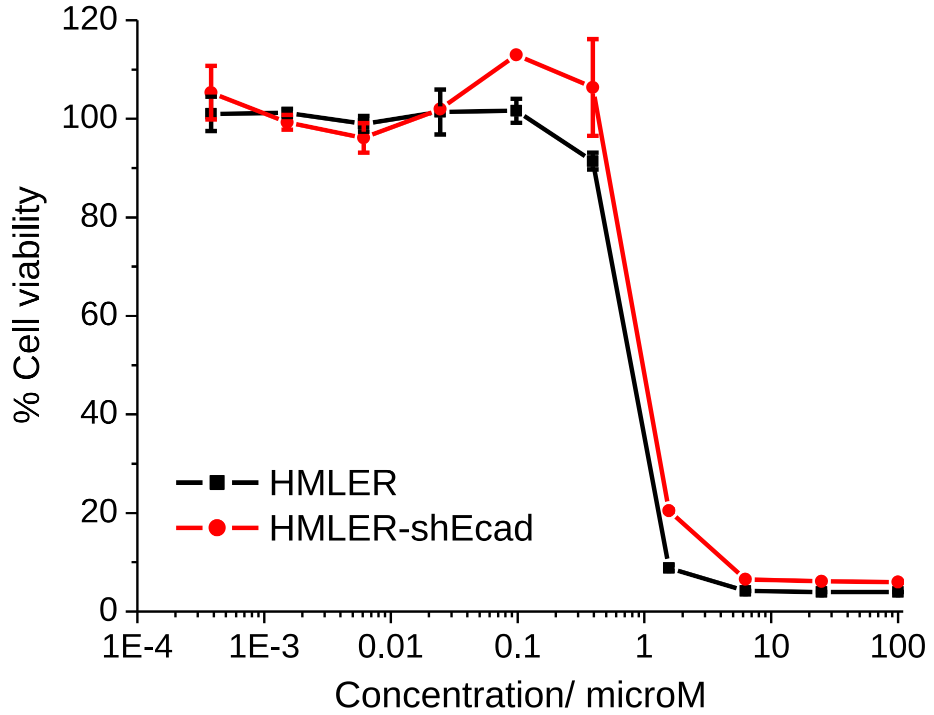

Figure S10. Representative dose-response curves for the treatment of HMLER and HMLER-shEcad cells with **2** after 72 h incubation.

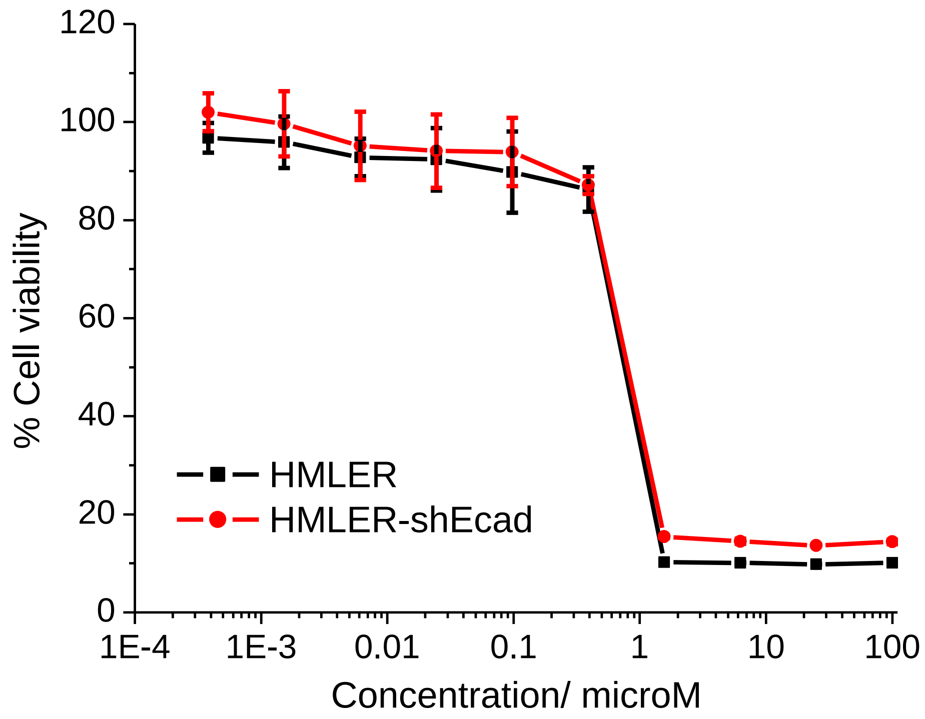

Figure S11. Representative dose-response curves for the treatment of HMLER and HMLER-shEcad cells with **3** after 72 h incubation.

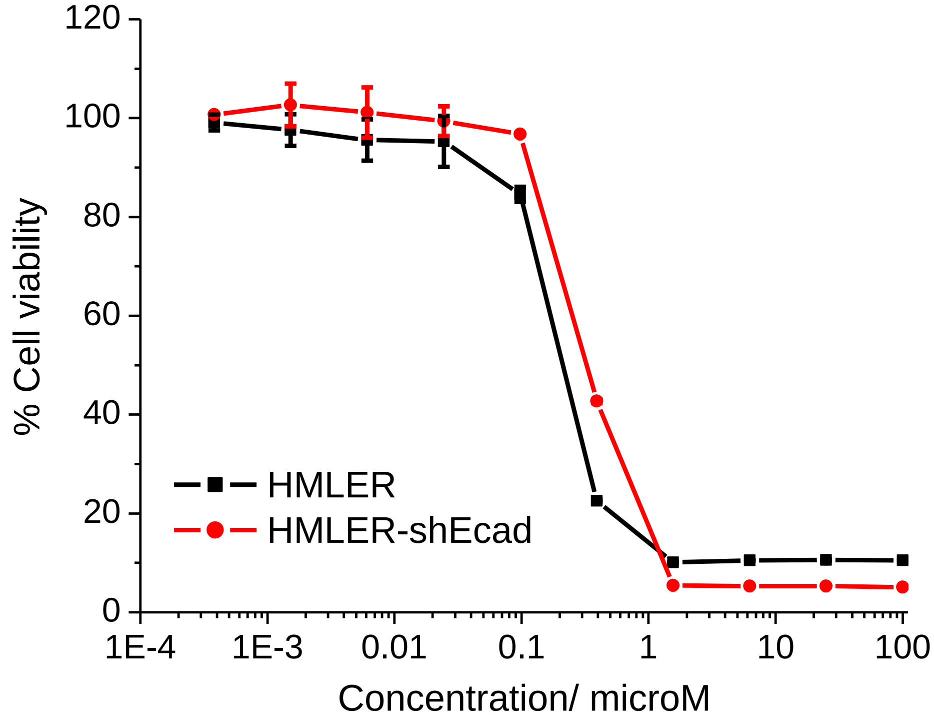

Figure S12. Representative dose-response curves for the treatment of HMLER and HMLER-shEcad cells with **4** after 72 h incubation.

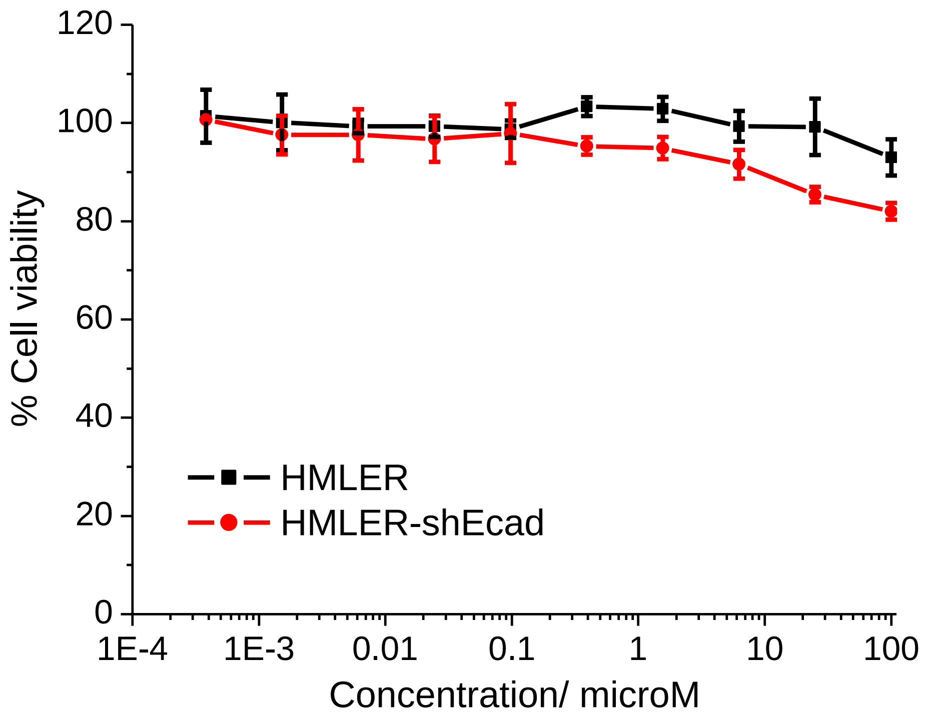

Figure S13. Representative dose-response curves for the treatment of HMLER and HMLER-shEcad cells with [Cu(**L**[1])Cl] after 72 h incubation.

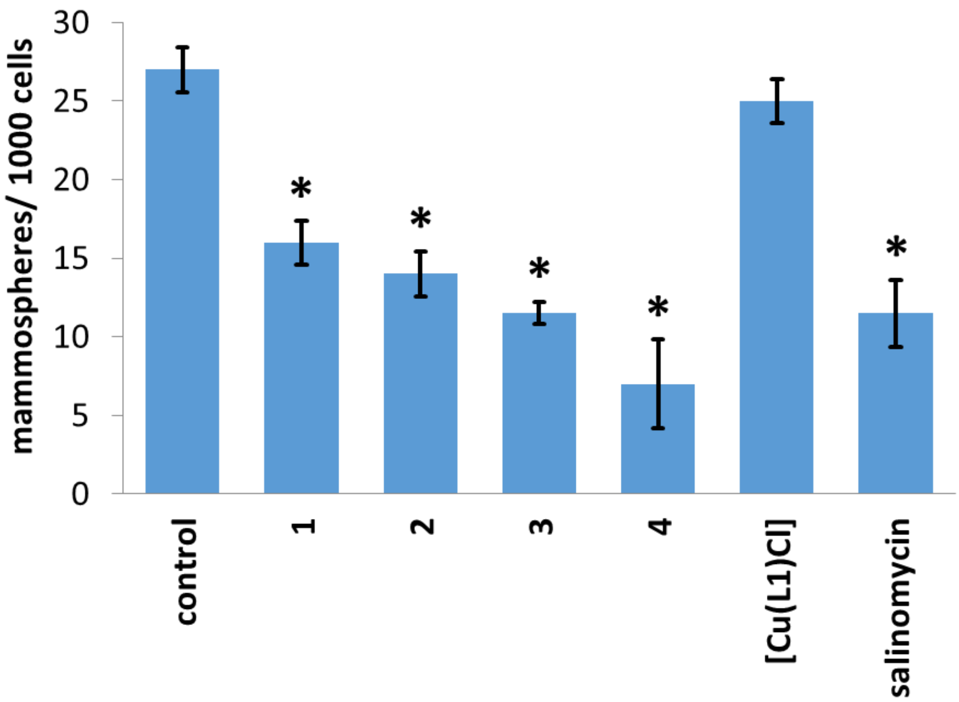

Figure S14. Quantification of spheroid formation with HMLER-shEcad cells untreated and treated with **1-4**, [Cu(**L**[1])Cl], or salinomycin at their respective IC_{20} values for 5 days. Error bars = SD and Student t-test, * = $p < 0.05$.

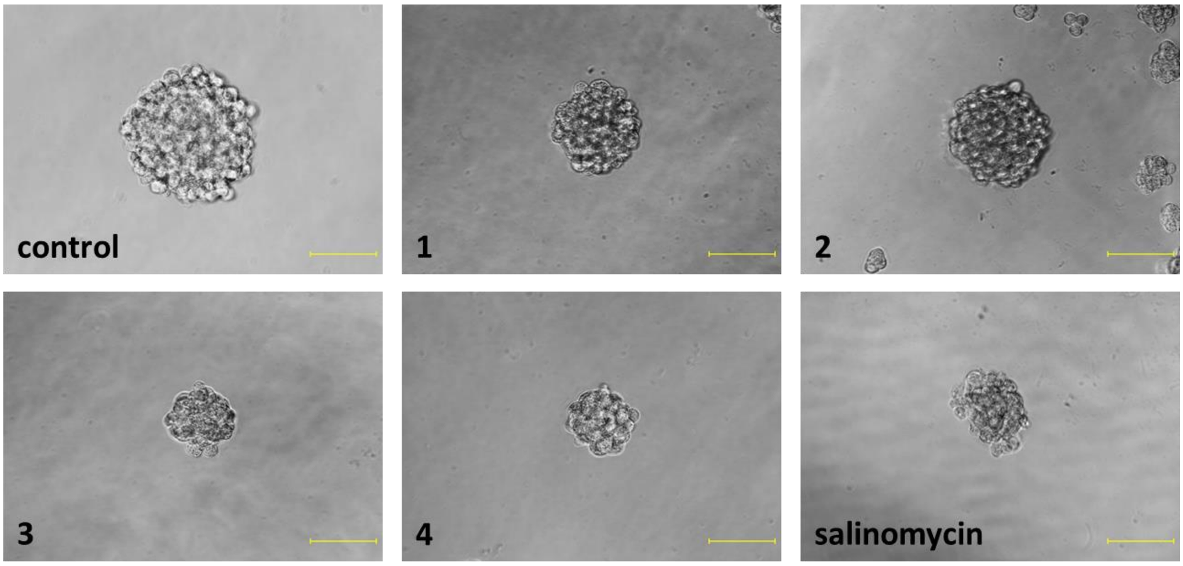

Figure S15. Representative bright-field images (× 20) of HMLER-shEcad spheroids in the absence and presence of **1-4** or salinomycin at their respective IC_{20} values for 5 days.

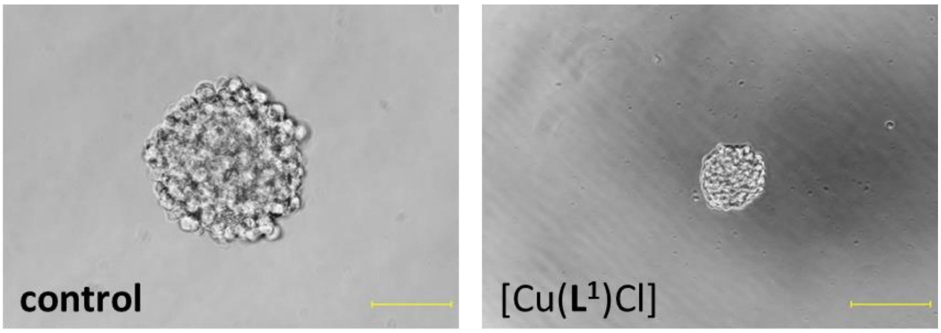

Figure S16. Representative bright-field images (× 20) of HMLER-shEcad spheroids in the absence and presence of [Cu(**L**^{1})Cl] at the respective IC_{20} values for 5 days.

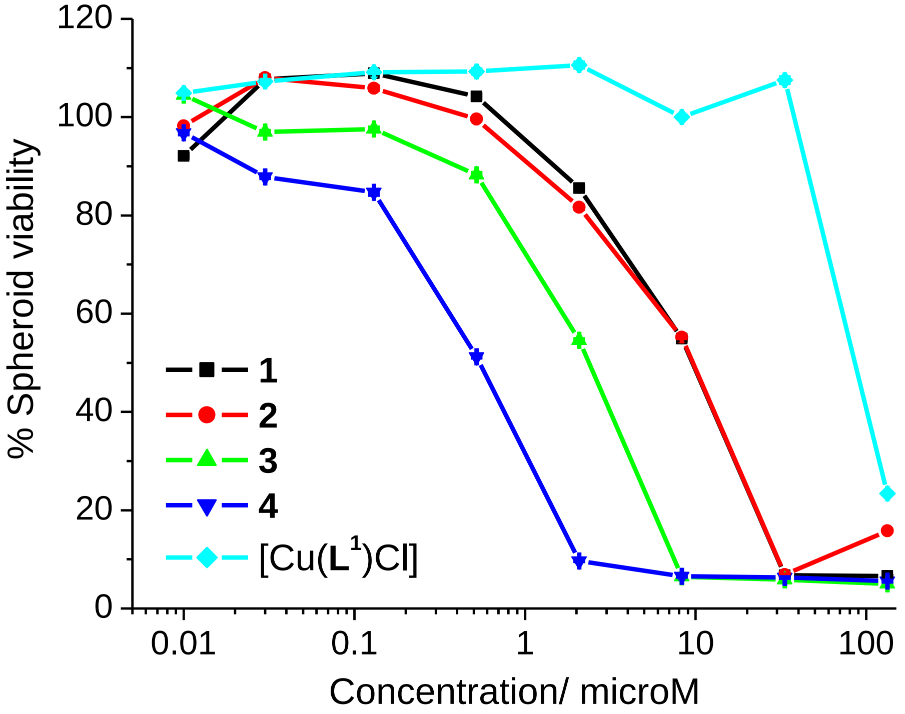

Figure S17. Representative dose-response curves for the treatment of HMLER-shEcad spheroids with **1**-**4** and [Cu($\mathbf{L}^1$)Cl].

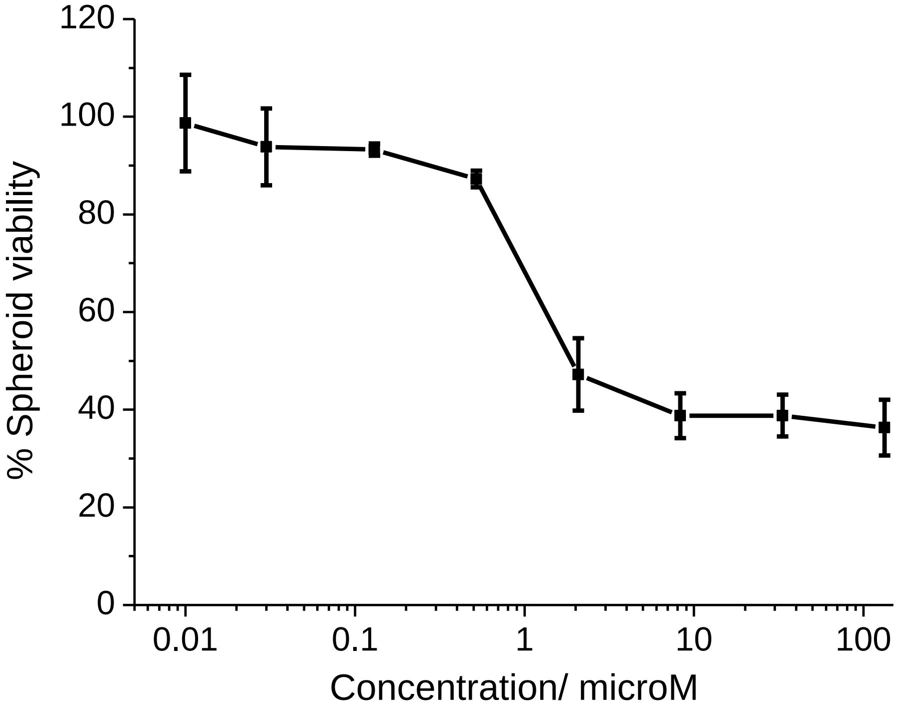

Figure S18. Representative dose-response curve for the treatment of MCF10A spheroids with **4**.

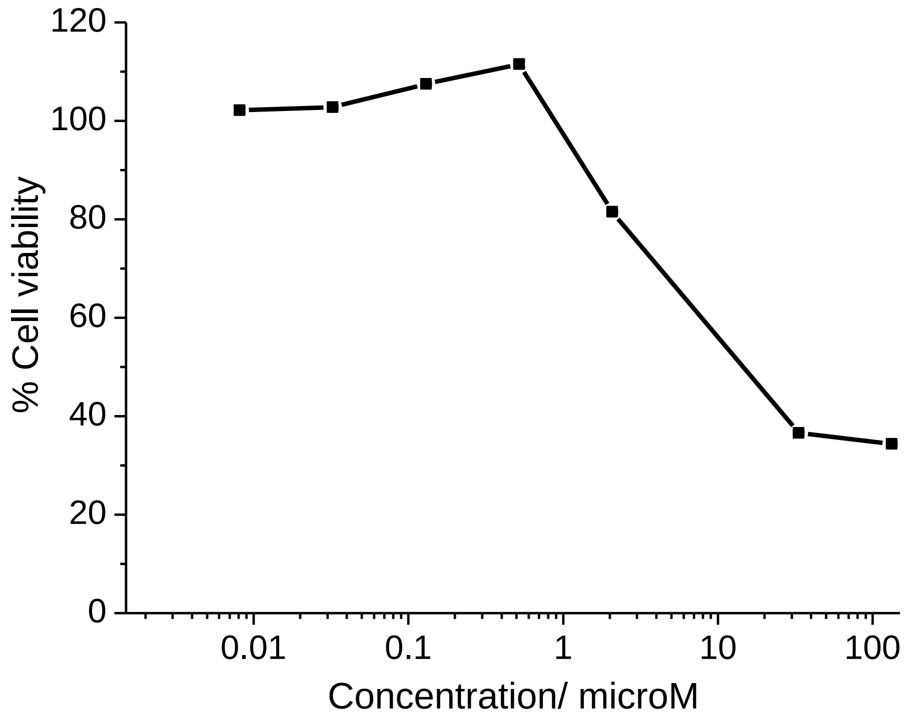

Figure S19. Representative dose-response curve for the treatment of MCF10A spheroids with salinomycin. Taken from reference [3].

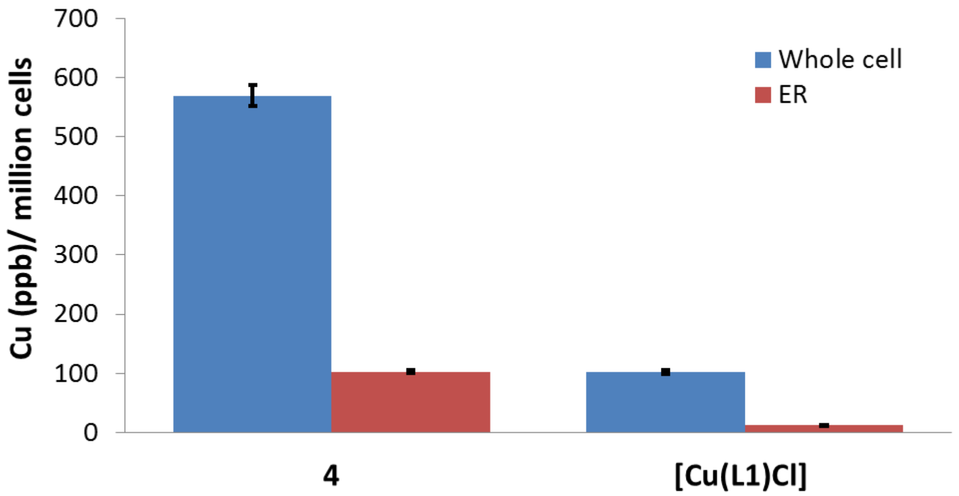

Figure S20. Copper content in whole cell and endoplasmic reticulum fractions isolated from HMLER-shEcad cells treated with **4** or [Cu($\mathbf{L}^1$)Cl] (5 µM for 24 h at 37°C). Error bars = SD.

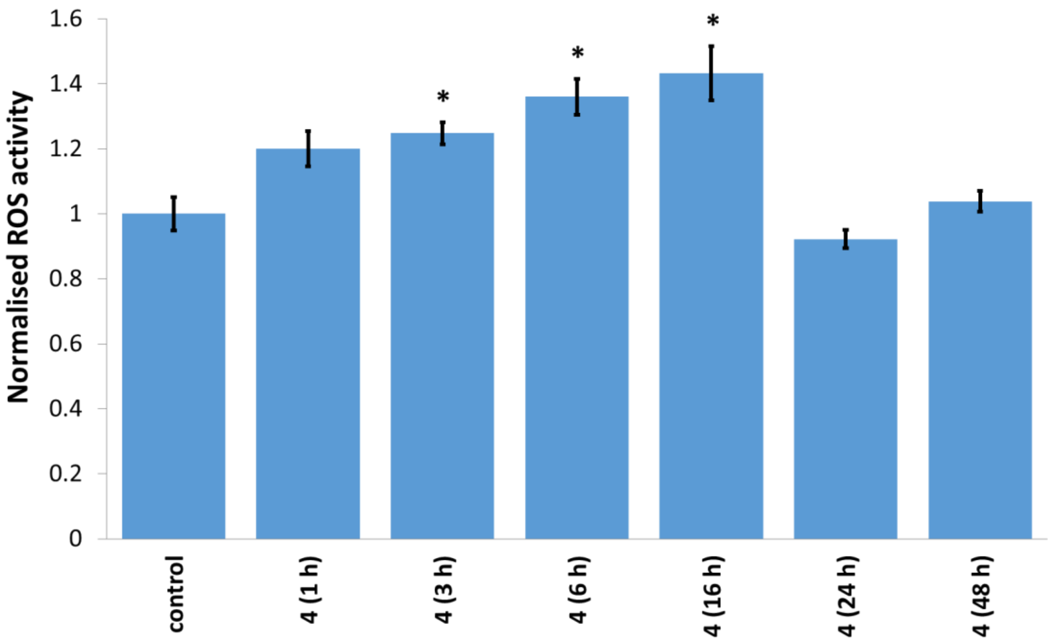

Figure S21. Normalised ROS activity in untreated HMLER-shEcad cells (control) and HMLER-shEcad cells treated with **4** (IC_{50} value for 1, 3, 6, 16, 24, and 48 h). Error bars = SD and Student t-test, * = $p < 0.05$.

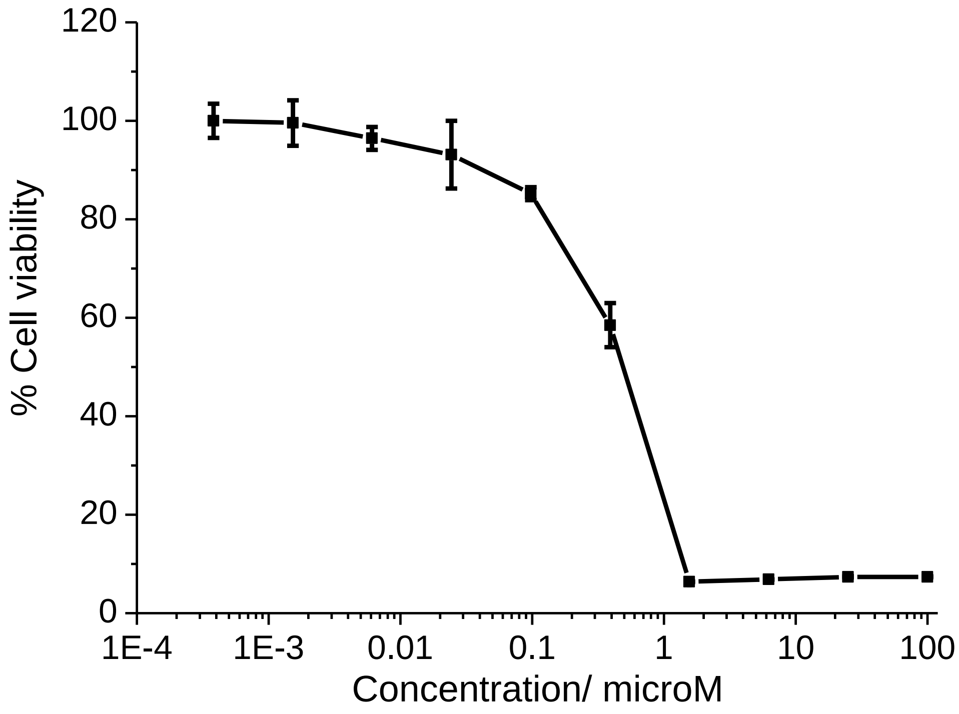

Figure S22. Representative dose-response curves for the treatment of HMLER-shEcad cells with **4** after 72 incubation in the presence of *N*-acetylcysteine (2.5 mM).

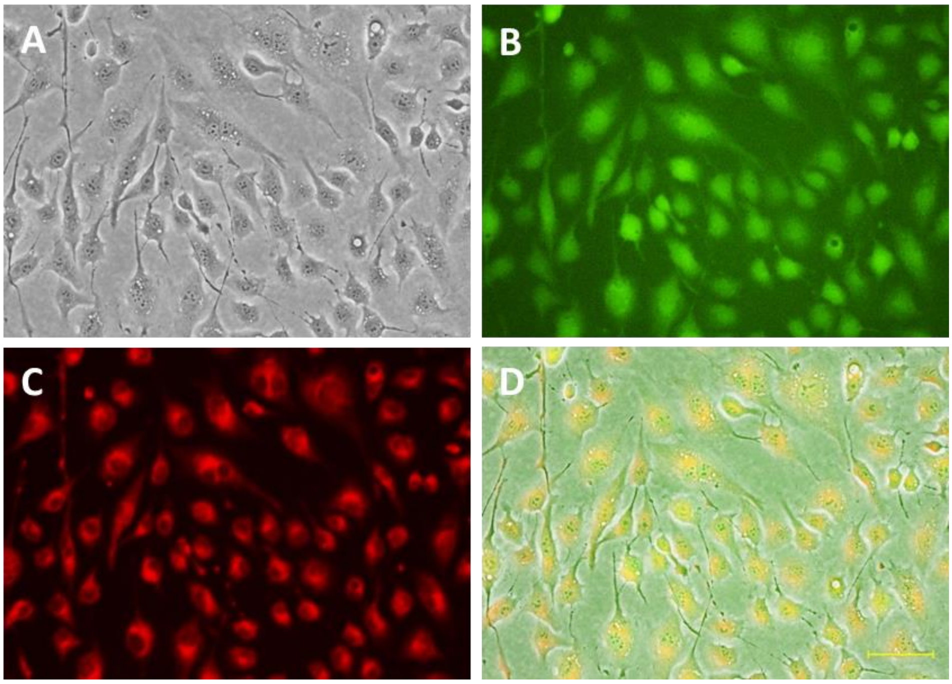

Figure S23. Fluorescence microscopy images (× 20) of live HMLER-shEcad cells co-treated with **4** (5 μM for 1 h), ER-Tracker Red (1.6 μM for 15 min), and green-emitting DCFDA (20 μM for 10 min). (A) Bright field transmission image (B) green channel image (C) red channel image, and (D) overlaid bright field transmission, green channel, and red channel image.

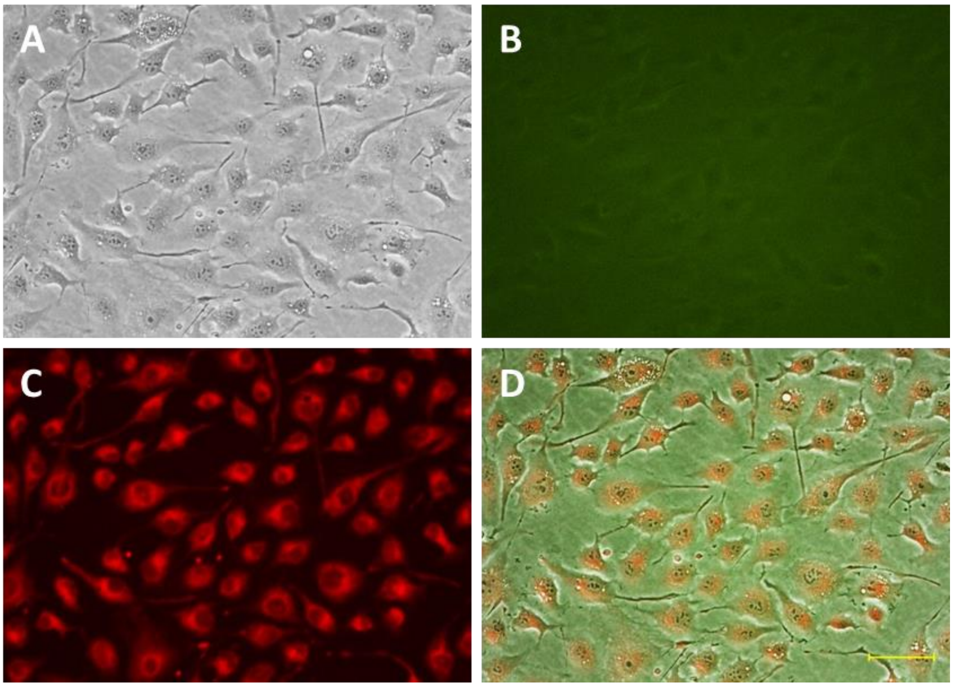

Figure S24. Fluorescence microscopy images (× 20) of live HMLER-shEcad cells co-treated with ER-Tracker Red (1.6 μM for 15 min) and green-emitting DCFDA (20 μM for 10 min). (A) Bright field transmission image (B) green channel image (C) red channel image, and (D) overlaid bright field transmission, green channel, and red channel image.

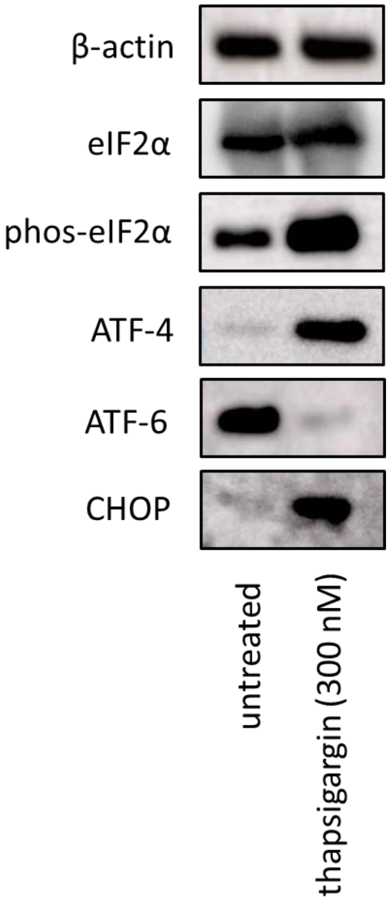

Figure S25. Immunoblotting analysis of proteins related to the unfolded protein response (UPR). Protein expression in HMLER-shEcad cells following treatment with thapsigargin (300 nM for 1 h).

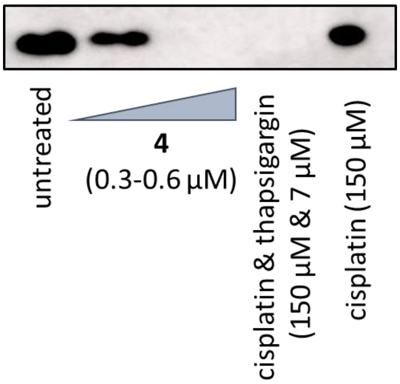

Figure S26. Immunoblotting analysis of high mobility group box 1 (HMGB-1). Protein expression in HMLER-shEcad cells following treatment with **4** (0.3 and 0.6 μM for 48 h) or cisplatin (150 μM for 48 h) and thapsigargin (7 μM for 48 h), or cisplatin (150 μM for 48 h).

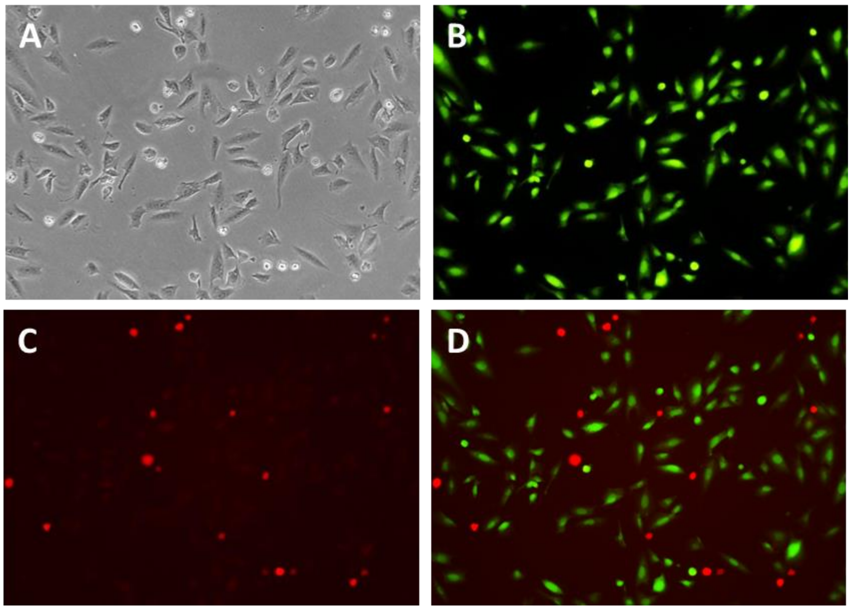

Figure S27. Fluorescence microscopy images (× 10) of CellTracker Green-stained HMLER-shEcad cells treated with cisplatin (50 μM for 4 h), and then incubated with CellTracker Orange-stained macrophages for 2 h. (A) Bright field transmission image, (B) green channel image, (C) red channel image, and (D) overlaid green channel and red channel images.

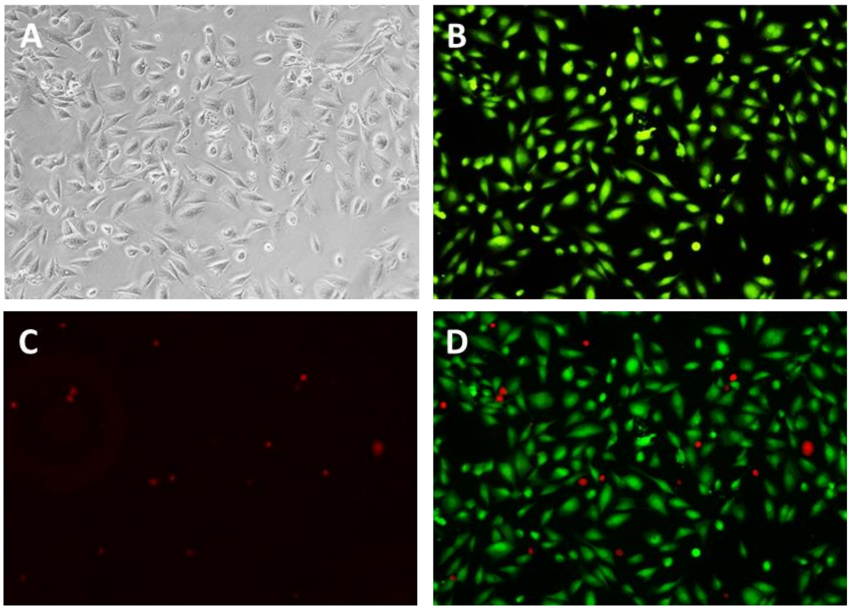

Figure S28. Fluorescence microscopy images (× 10) of CellTracker Green-stained HMLER-shEcad cells treated with carboplatin (100 μM for 4 h), and then incubated with CellTracker Orange-stained macrophages for 2 h. (A) Bright field transmission image, (B) green channel image, (C) red channel image, and (D) overlaid green channel and red channel images.

References

[1] a) C. Lu, A. Eskandari, P. B. Cressey, K. Suntharalingam, *Chem. Eur. J.* **2017**, *23*, 11366-11374; b) S. Dhar, A. R. Chakravarty, *Inorg. Chem.* **2003**, *42*, 2483-2485.

[2] G. Sheldrick, *Acta Cryst.* **2008**, *A64*, 112-122.

[3] A. Eskandari, K. Suntharalingam, *Chem. Sci.* **2019**, *10*, 7792-7800.
